# A Multiple Case Study of Mental Health Interventions in Middle Income Countries: Considering the Science of Delivery

**DOI:** 10.1371/journal.pone.0152083

**Published:** 2016-03-24

**Authors:** Sean A. Kidd, Athena Madan, Susmitha Rallabandi, Donald C. Cole, Elisha Muskat, Shoba Raja, David Wiljer, David Aylward, Kwame McKenzie

**Affiliations:** 1 Department of Psychiatry, University of Toronto, Toronto, Ontario, Canada; 2 Toronto Centre for Addiction and Mental Health Toronto, Ontario, Canada; 3 Dalla Lana School of Public Health, University of Toronto, Toronto, Ontario, Canada; 4 Ashoka Canada,Toronto, Ontario, Canada; 5 BasicNeeds,Bangalore, India; 6 Ashoka, Washington D. C., United States of America; University of Connecticut, UNITED STATES

## Abstract

In the debate in global mental health about the most effective models for developing and scaling interventions, there have been calls for the development of a more robust literature regarding the "non-specific", science of delivery aspects of interventions that are locally, contextually, and culturally relevant. This study describes a rigorous, exploratory, qualitative examination of the key, non-specific intervention strategies of a diverse group of five internationally-recognized organizations addressing mental illness in middle income countries (MICs). A triangulated approach to inquiry was used with semi-structured interviews conducted with service recipients, service providers and leaders, and key community partners (N = 159). The interview focus was upon processes of implementation and operation. A grounded theory-informed analysis revealed cross cutting themes of: a holistic conceptualization of mental health problems, an intensive application of principles of leverage and creating the social, cultural, and policy “space” within which interventions could be applied and resourced. These findings aligned with key aspects of systems dynamic theory suggesting that it might be a helpful framework in future studies of mental health service implementation in MICs.

## Introduction

In a concerted effort to address the large and growing burden of mental illness in low and middle income settings (LMICs), the field of global mental health (GMH) is increasingly focussed upon the application and scaling up of evidence based interventions, with an emphasis upon task shifting [1]. Considerable controversy attends this emphasis upon standardization and scaling, with randomized trials as the gold standard of evidence [2]. There is a concern that Western, biomedical conceptualizations of mental illness and treatment can pathologize the individual and that the focus should be on the social determinants of illness. The critique goes further to note that this emphasis does not address non-technical/specific aspects of intervention and important local, contextual and cultural factors. The dialectic in this debate suggests that the greatest impact and ethical rigour in GMH requires a balanced consideration of the benefits of scaled, evidence-based approaches, particularly for severe mental illness while at the same time carefully attending to importance social determinant, cultural and contextual processes [2].

This debate may partly grow out of an imbalance in the base of evidence. Specifically, a considerable amount of evidence is available regarding assessment tools and protocols for an array of brief interventions for mental illnesses in LMICs [3]. Such approaches readily lend themselves to conventional clinical research designs. Far less information is available regarding what are variably referred to as the non-technical or non-specific intervention and service process-oriented factors that many would argue are essential to generating substantive impact [4]. In the broader healthcare arena this has been referred to as the "science of delivery"[5]. The problem that arises is that the more circumscribed, trial-amenable interventions cannot succeed without attention to the quality of the service processes and contexts in which they are implemented [6].

Social entrepreneurship is one lens that is available for understanding what will henceforth be referred to as "non-specific" factors such as models of leadership, partnership, and context-relevant conceptualizations of mental illness and intervention [7,8]. Social entrepreneurs have been characterized as change agents who utilize highly flexible approaches to solving social problems that allow them to effectively bridge gaps between multiple sectors and systems [9–11].

Cultivated to a large extent by organizations such as Ashoka [12] and the Skoll Foundation [13], social entrepreneurship has emerged as a prominent way of thinking about how complex problems, social and otherwise, are addressed. Health has been regularly connected with social entrepreneurship, with a recent review of 366 case studies finding that health was the primary target in 18% of the cases [14]. Social entrepreneurship in mental health specifically has had less attention despite its relevance in addressing the complexity of mental illness in under-resourced contexts [7].

This paper describes a rigorous examination of the key, non-specific or delivery system intervention strategies of a group of internationally recognized organizations addressing mental illness in middle income countries (MICs)—all of which have been identified as leading examples of social entrepreneurship. It is intended to inform the conversation about balance in considering how interventions are best developed and scaled in under-resourced contexts. It is among the first to apply a rigorous research design in this less well-defined domain of service delivery—studying the organization and service delivery models through which interventions are optimally deployed.

## Methods

### Case Identification

This study employed an instrumental, multiple case study design, one in which case study findings are used to inform the understanding of a specific broader issue [15]. The organizations that were studied were founded by Ashoka Fellows. Ashoka is the organization that is arguably the most prominent, internationally, in employing a social entrepreneurship lens to identify some of the most promising individuals and organizations that address social problems [12]. While Ashoka’s network by no means represents all social entrepreneurs, it is known for its accuracy in identifying highly effective social entrepreneurial approaches. It uses an intensive Delphi method [16] to identify potential Fellows and applies a rigorous selection process with a panel review that attends to key domains of social entrepreneurship, impact, and scale or potential for scaling. The Ashoka website at the time of inquiry described the work of 2,663 Fellows and their organizations from over 70 countries. The Fellows working in mental health were identified through (i) a keyword search of the online Ashoka directory using the terms: “mental health”, “mental illness”, “psychiatric”, “addiction”, and “developmental”; and (ii) cross-referencing the list with Ashoka staff familiar with the Fellows working in health to determine if any had been missed. This two-part process revealed 42 Fellows. Detailed descriptions of all 42 Fellows were reviewed by (Cole, Kidd, McKenzie, and Wiljer) at the level of their Ashoka profiles with further inquiry through websites, publications, and with Ashoka staff (e.g., to ensure the organization is still active). A maximum variation sampling strategy was used to identify 5 Fellows and their organizations with which to engage in intensive case studies. Target problem (type of mental health concern), intervention type/approach (service model; scale and reach), target population (by age, rural/urban, socioeconomic status), and geographic location (sociopolitical context past and current; culture) were key dimensions considered in achieving adequate variation, which was an important consideration given the intent to identify broadly applicable themes.

Given the depth of inquiry it was anticipated that 5 case studies would prove adequate to inform the service implementation and process questions that are the focus of this study and to reach saturation in the analysis [17]. The study was reviewed and approved by the Toronto Centre for Addiction and Mental Health Research Ethics Board. The protocol was also reviewed and approved by the executive leadership and research leads (individuals responsible for research within the organization, ethics review and approval) of each of the participating organizations. None of the organizations approached refused participation and the five that participated were the five originally selected.

### Data Collection

Contextual information regarding organization operations and settings were collected through documentation review including websites and annual reports. This review also assisted with tailoring the plan of inquiry for the specific organizations. The primary source of data was semi-structured interviewing with past and present service/organization leadership anddirect service staff with service recipients and key partners (often less aware of implementation and process details) acting as secondary, triangulating sources of information. Key partners included policy makers who had been regular contacts and practitioners associated with partner organizations as identified by case study organization leadership. Service recipients were approached upon the recommendation of staff who assisted with addressing questions of capacity and minimizing intrusiveness in service settings. Leaders and staff had been informed of the intent to connect with stakeholders ranging in degree of supportiveness of their work. Nonetheless, it is possible that some bias might have attended this method of recruitment. The study also included ethnographic observation of activities of the organization (e.g., non-clinical meetings of staff and clients (e.g., in community activities, common spaces), presentations to the public, advocacy activities, etc.) with observations recorded in field notes. The ethnographic component occurred primarily during tours of organization activities by staff as well as interviewers spending time in common spaces. Interviewers were introduced as researchers attempting to develop an understanding about how a given organization worked. Multiple interviews were undertaken with service leaders and their staff, providing opportunities to ask follow up questions as the inquiry and data analysis progressed. Interviews with other stakeholders took place on a single occasion. Interviews were audio-recorded and transcribed verbatim, with translation services used on an as-needed basis, and took place between May, 2014 and March, 2015. Written consent was obtained for interviews with service leaders, staff, and key partners. Verbal consent was obtained from service recipients due to literacy difficulties in the study contexts, and was documented by noting the name, date, and location in which consent was secured. No service recipient was included for whom there was any question of capacity or any other consideration that might affect their understanding of their involvement. Interviews with service recipients were designed to be minimally intrusive, focusing on perceptions about what had been helpful or less helpful in their service experience over time. This verbal consent procedure was approved by the Research Ethics Board. Interviews were conducted by postdoctoral fellows (Madan and Rallabandi) who had no prior relationships or conflicting interests with the organizations of focus.

Specific areas of inquiry included a detailed history tracking the trajectories and turning points in services offered, service structures/models, goals and values, implementation, leveraging support, and relationships with stakeholders and supporters (See S1 Appendix for interview protocols). We undertook an examination of the specific aims and activities of the people involved in the service. Implicit to this line of investigation was a close study of the “theories of action” of the participants–their understanding of how their activities lead to the outcomes that they are seeking to attain. Also examined were their relationships within relevant systems (service, corporate, government, cultural norms)–how they are positioned, how initiatives are achieved or blocked, and the flow of information, people, and resources into and out of the organization from these systems. This attention to the ‘circumstances’, structures, processes, and contexts of the interventions has been previously highlighted as essential in such case studies [18].

### Data Analysis

Following Stake’s model for multiple case study analysis [19], we analyzed transcripts and field notes using a thematic analysis informed by grounded theory (i.e., employing constant comparison, exploring connections between themes to inform a more theoretical formulation) [20]. The analysis moved from line-by-line open coding, to the refinement of codes and the development of an overarching thematic framework. Data collection and analysis proceeded simultaneously, allowing for opportunities to refine structure of the findings through increasingly targeted inquiry.

Several steps were taken to maximize rigor in the analysis and to establish the credibility and trustworthiness of the findings. First, there was a very rich set of data in multiple forms that allows for triangulation by source (staff, leaders, partners, observation) and type (field notes, verbal description). Second, participants were engaged in discussion about the emerging categories to support trustworthiness in the analysis. Third, a method of employing multiple coders was undertaken to develop the thematic framework with multiple rounds of review (by Kidd, Madan, and Rallabandi), negotiation of different perspectives, and revision.

## Results and Discussion

### Participating Organizations and Individuals

The five organizations selected for study were BasicNeeds and its sites in Vietnam, and Ghana [21], the Acid Survivor's Foundation in Bangladesh [22], the Banyan in Chennai, India [23], ADVANCE in Egypt [24], and the Fundacion Colectivo Aqui y Ahora in Colombia [25] Table 1. All of these organizations have mental health as a focus (though have a holistic approach that considers physical health, social and cultural domains) and operate where such supports had previously been extremely limited or completely lacking. BasicNeeds is among the largest and most extensively scaled comprehensive interventions to address mental illness globally—with 12 sites and over 600,000 beneficiaries to date. In Ghana and Vietnam, BasicNeeds operates out of several sites, both rural and urban, targeting impoverished people with mental illness. The Acid Survivors Foundation, which is based in Dhaka but has a national reach, provides a diverse set of services from plastic surgery to psychosocial rehabilitation for Bangladeshi people who have survived acid violence. The Banyan provides an intensive array of services for homeless individuals with severe mental illness with the core services studied located in Chennai. ADVANCE in Egypt provides specialized services for children and adolescents with autism spectrum disorders to support improved social integration. ADVANCE operates a stand along education and support centre and provides a range of outreach supports in close connection with schools and parent groups. The Fundacion Colectivo Aqui y Ahora in Colombia addresses youth addictions through a methodology focused upon personal meaning with engagement at family, school, workplace, and public levels. It has a standalone clinic and has outreach activities taking place at a national level in the range of settings noted above. Further details regarding the operations of each of these organizations and the Ashoka Fellows associated with them can be found at the referenced websites or at www.ashoka.org.

**Table 1:** Organization Descriptions.

Organization Name and Target Population	Location(s)	Scope of Service (all engage at policy levels, education and awareness raising)	Formally Launched and Associated (Ashoka Fellow)
BasicNeeds (Individuals with Severe Mental Illness)	UK (home office), China, Ghana, India,Kenya, Lao PDR,Nepal, Pakistan,South Sudan, Sri Lanka, Tanzania,Uganda,Vietnam	Scaled and franchised treatment model that provides medication and psychosocial support in partnership with local governments. Incorporates self-help groups and social enterprise.	2000 (Chris Underhill)
Acid Survivor’s Foundation (Acid Violence Survivors)	Bangladesh	Operates a 20-bed hospital providing burn care services, psychological care, legal assistance and financial support.	1999 (Monira Rahman)
The Banyan (Homeless Persons with Mental Illness)	India	Provides a range of supports including medication, psychological, vocational, and occupational interventions. Housing supports also provided.	1993 (Vandana Gopikumar)
ADVANCE (Children with Autism)	Egypt	Offers a multidisciplinary therapeutic program focusing on cognitive and skill development. Includes speech and language therapy and psychomotor therapy.	1997 (Maha Helali)
Fundacion Colectivo Aqui y Ahora (Youth with Addictions)	Colombia	Employs a holistic drug treatment and prevention model in school, family, and workplace contexts that focusses on the development of personal meaning	1997 (Efren Martinez)

The total number of individual participants interviewed was 159 with a mean of 31.8 interviews per organization across the 5 case studies. Broken down by type, this included 93 service/organization staff and leadership, 46 community collaborators and members with a range of connections with the organization, 22 individual service recipients and 7 self-help groups with memberships of up to 30 people (for interview details see Table 2). These distinctions are somewhat artificial, as in these organizations there are many examples of service recipients becoming staff. Variability in interview and participant times were accounted for by a number of factors including opportunity with respect to time and travel, availability of participants, and sensitivity with respect to some groups of potential participants (e.g., children with autism). Interviews ranged in length from 15 minutes in a single contact (typically community members and recipients) to over 6 hours over multiple contacts (typically with service leaders and staff). Overall, the interview content was rich though in some instances it appeared that histories of conflict and other sociopolitical factors may have influenced the depth of inquiry (e.g., fears of discussing or criticizing systemic barriers in contexts where such talk in the past or present could lead to severe reprisal).

**Table 2:** Interview Details by Case Study Site.

Site	Total Number of Interviews	Staff/Leaders n (mean years; range)	Key Partners n	Beneficiaries n	Self Help Group Interviews
Acid Survivor’s Foundation	25	20 (6.4;1–16)	5	2	0
Fundacion Colectivo Aqui y Ahora	30	14 (5.19;1–18)	11	5	0
ADVANCE	19	17 (10.2; 2–19)	2	0	0
Banyan	19	17 (9.3;1–23)	2	0	0
BasicNeeds (UK and India Admin Offices)	12	12 (9.1;2–15)	0	0	0
BasicNeeds (Vietnam)	39	11 (7.0;3–13)	16	12	2
BasicNeeds (Ghana)	15	2 (9.0;0.5–27)	10	3	5

### Overview: Leverage and Space in Which to Work

Despite the considerable diversity across the specific operations and contexts of these organizations, a saturated and coherent theme structure readily emerged in the data analysis. The two cross-cutting thematic elements were leverage and the generation of resources (e.g., social capital) to support the development, capacity, and scaling of the interventions. Here, leverage could be considered the effort to maximize the outputs of every available resource, while applying pressure to aspects of the given complex problem in a manner that results in the maximum amount of change. Concurrently, these organizations ambitiously and persistently created space in which to work—with space being considered the cultivating of public, political, social and cultural resources around the problem. This provided social and fiscal capital and systems in which bureaucracy and policy enabled rather than hindered efforts. These core themes and their subthemes are described in detail below along with full quotes and excerpts that are representative of the data from which they were derived.

### Leadership

*Understanding people*, *treating people equally*, *being non-judgmental*, *being down to earth*, *open minded*, *balanced between the rational and emotional*, *a strategic thinker*, *a good communicator*, *trustworthy*, *setting examples by doing things*, *team building*, *team spirit*, *mobilizing people and resources*.

(Past Executive Director)

This quote concisely encompasses most of the elements that participants considered important in the leadership of these organizations. They were described as individuals with a specific set of skills and values. They are intimately familiar with and intensely, personally invested in the cultures and contexts in which they work.

*"Initially*, *though they were the founders*, *they didn’t mind doing anything*. *They have cleaned the bathrooms*, *they mopped the floors*, *and they used to cook in the kitchen*, *they used to clean and wash the clients*, *they themselves went for rescues*, *they themselves used to serve food*. *They have done the maximum from the beginning*. *They have done so many things from the ground level and that made them to grow higher and earn so much of manpower through the services that they have rendered to the clients*."

Direct service staff person

Whether directly affected by mental illness in their families or through a very intensive study of the issues involved through "sitting with, living with" and "supping with" clients, families and communities, this empathetic approach had numerous implications. It was described as leading to an extremely deep understanding of the problem that needs to be addressed, the contexts and dynamics associated with it, and an intense engagement with and knowledge about the relevant stakeholders. This base of knowledge fed into, without exception, an understanding that effective interventions need to be individualized, staged, and multifaceted to address the many interacting needs of clients, families and communities. Furthermore, in the complex systems that surround the mental health problem that they sought to address, such a depth of knowledge and engagement allowed them to better recognize opportunities, resources, and points of leverage.

*“So*, *as parents we got together and we had parents support meeting and we decided that what our children need is beyond what a center can do*, *we need a total NGO*, *we need life span services*, *we need awareness*, *we need advocacy*, *we need a lot of other things that’s not there*. *We also need to train personnel*, *because the personnel who existed knew maybe about disability but were not specific in their knowledge about autism and sensory issues and so on*.”

Executive Director and Founder

This depth of understanding was complemented by skill as educators and communicators who "cultivate trust" and "respect" in aligning diverse stakeholders around a clear vision of the problem and its solution–whether person with mental illness, rural villager, senior policy maker or influential public figure. This knowledge and skill in engaging and aligning had clear implications for the core theme of creating space in which to work as access and support across sectors requires this type of alignment.

Leverage, in the form of maximizing everyone’s potential contribution, was evident in their ability to "diagnose the efficiency and hidden talent" of clients, volunteers, and staff. All described working in low-hierarchy environments where everyone was encouraged to explore and extend the limits of their capabilities. In commentary about process over time, while the launch of these endeavours were typically framed as a "passionate outburst" in addressing a human rights issue, over time strategy became much more of an emphasis. For example, one core strategy was to innovate and expand through experimentation ("low cost, low risk experiments") while skillfully advancing large and complex organizations, carefully assessing points of leverage, timing, and maintaining fidelity to the intervention and its values.

### Livelihoods

Attention to livelihoods was a pivotal part of this understanding of client needs across interventions. It was discussed as a leveraged part of the mental illness problem for two reasons—one is that mental health interventions are much less effective without attention to livelihoods. Treating mental illness is "secondary to survival", and people fear the costs, financial and otherwise, of engaging in treatment.

*“I was 22 years old and had mental illness and nobody understood what was wrong with me and it shamed my parents*, *I was unmarriageable and a burden to their house*.”

Service Recipient

Conversely, attending to livelihoods had many advantages—ranging from the direct benefits of being more active to addressing stigma. Working unlocks sources of support from family and communities and through contact with community reduces isolation and social stigma—demonstrating that people with mental illness can contribute and creating opportunities to improve impacts and expand.

*“We saw her progress*. *And that she could do this [broommaking]*. *It was satisfying seeing her return to find a useful purpose*. *So she is not forgotten in society*. *She makes brooms and with that*, *has money and earnings so that she can buy what she needs to eat*. *She can go out and participate in community*, *at pagoda*. *She is participating in meetings with her brooms—this helps relieve stress and deal with thoughts and be part of community life*. *The illness is managed/decreased and gradually her health is better*.”

Family Member

### Empowerment

These interventions were described as fundamentally empowering—this is true for staff and volunteers as described above but emphasized to an even greater extent with respect to service recipients. As with the themes above this had clear implications for leverage in interventions and enhancing reach. The use of the term empowerment by the participants in these case studies was very well aligned with the definition of prominent consumer-survivor advocate Dr. Pat Deegan who described a taking back of power "to become sovereign over our own lives and bodies, to reclaim our right to make choices and have access to resources to improve the quality of our lives."([26], p.11) Grounded in empathetic and rights-oriented approaches, these organizations described very actively seeking out and cultivating the "inner strength" of clients.

*"These survivors at the beginning were completely hopeless and helpless*…*they were not even thinking about surviving*. *They were thinking about committing suicide*. *So when the survivors found that someone is understanding them*, *treating them as real human being*, *respecting them*, *respecting their values and their thoughts and helping them to express themselves*…*that actually helped them more and more*. *So*, *when [acid violence survivor] started to talk with other survivors who were receiving treatment in Dhaka medical college hospital*, *in that way*, *drop by drop they were also growing*."

Service Staff Member

Many benefits of an empowering approach were described. These included the exposure of communities to empowered people with mental illness, which reduced stigma and increased expectations for what they might accomplish in life. This empowering approach also benefited an array of advocacy and fundraising activities—not in the form of narratives of people being "used" to raise funds, but of organizations creating spaces where people could realize their full potential and make a difference.

“*This aspect of their work*, *the empowerment piece*, *was described by our participants as one of the most rewarding*. *It energized*, *inspired*, *and is "the heart of the work*."

Direct Service Staff Member

Another thread involved the quality and availability of services. Peer support, developed in these contexts of empowerment, was very actively utilized as a leveraged strategy. It was clear in the narratives of peers, those receiving peer support, and staff, that the employment of peers further cultivated messages of hope and empowerment. Peers worked in ways fundamentally grounded in lived experience knowledge about mental illness, along with a deep knowledge of relevant community resources and local cultures. Peers were described as essential to expanding the reach and impact of these organizations.

*… ‘We feel this work is better done by us because it is we who are sick and should therefore support each other… Village mobilisers are more familiar with the participants in their community*, *have a vested interest in the project*, *have experience of treatment and are aware of the benefits of the project to participants’ lives*.”

Peer Provider

### Directly Engaging and Involving Key Stakeholders

Along with the more obvious financial capital necessary to operate, the participants spent a considerable amount of time discussing how the problem needs to become seen as important and social capital needs to grow around it.

*“We formed different advisory groups*, *the legal advisory group*, *the prevention advisory group and these people*, *the experts were helping us in developing strategies and mobilizing resources not just financial but also other resources and in that way*, *gradually we are making a public opinion against acid violence*. *I should say that one event which was a kicking point is like in 2002 on International women’s day*, *we organized men’s rally*. *That was organized in collaboration with BRAC and the daily newspaper*. *In the year 2000 they established a forum to help acid survivors*. *They were also mobilizing resources*. *In this Men’s March*, *over 5000 men participated and hundreds of female survivors were leading that March and media*, *national and international has publicized that issue*. *So*, *that created a huge movement in the country*.”

Organization Senior Staff

These organizations had cultivated relationships with a range of media outlets and created compelling stories about their work and the problems that they were addressing. Along with creating attention and interest among the general public, most were active in the engagement of key stakeholders whether through a media lens (e.g., supportive celebrity advocates) or with others who have influence. This extended as well into the realm of formal education—offering for free highly regarded trainings to government ministries, lawyers, police, and judiciaries on relevant issues and offering helpful practical advice and assistance. This work was further leveraged, through partnerships with and the involvement of lawyers, with the effort to strike down policies that posed barriers and enact helpful policy. Indeed, this active engagement of government was described by some leaders as essential to sustainability and scaling—even a prerequisite to partnership despite the time it can take to develop and the problem of policies being developed but not implemented in practice.

Branding was another key part of these conversations about creating space in which to work. The brand of the organization was established in several ways. They described cultivating an ethos, a set of values, and a distinctly innovative approach that operates in contexts where there would otherwise be little or no service. Respect was garnered through this way of working, as well as through the generation of impacts for large numbers of people who previously might have been considered hopeless by policy makers and the public.

*“We’re a very prominent part of the global mental health community*. *And we have a place there*. *It’s not a cakewalk—sometimes I get ignored around the table but we are starting to turn that around*. *People see us as a competent*, *collaborative presence that is considered an attractive partner to collaborate with*.”

Organization Senior Staff

Branding was also enhanced through connections from local to global levels with other prominent organizations, and by becoming well-known for their provision of high quality education opportunities to a range of sectors (e.g., government, other service providers, police). The benefits of cultivating such a brand had many advantages, including its feeding into more effective advocacy through access to public and policy forums, improved fundraising ability, and attracting and retaining skilled staff.

Participants also described facing the challenge that, unless they are able to successfully educate, raise awareness, and engage communities and families at a 'grass roots' level, interventions would not be accessed. As one community partner put the problem: "When I go to people's houses, they will let me come in, but I always hear ‘Come in for tea, okay. but don't do that stupid screening, we are fine." It was clear that "knowledge of the population" was crucial to turning this problem around—understanding how to engage diverse communities who may be facing poverty and have low educational backgrounds. This required persistence, but also an understanding of what is important to people—such as the enhancement of the livelihoods. Similar to the ways of engaging clients, the key theme of collaboration and respect were important here as well.

*"Strangely*, *we have faced the least challenges at the community level*, *particularly the rural communities have been very receptive and I think they are intelligent people*…*They are receptive because they are intelligent and they know the difference between somebody who is attempting to work with them and genuinely make a difference versus somebody who is exploiting them*, *somebody who is talking down or condescending*. *We learn a lot based on what the community tells us*, *we develop our services*. *So*, *some expertise we have*, *some expertise the community has*. *So*, *it is collaborative in that sense and I think they recognize that*."

Organization leader

Building collaborations with communities and families was not always described as reaching out—learning from and engaging communities and supporting clients in community based activities and advocacy. A big part of this effort involved *inviting people in*. This took place in a range of ways.

*"We cover five arrays in youth club*. *It is education*, *sports*, *culture*, *dance and vocation [programming available to the local community who join clients in these activities]*. *So*, *in education*, *we have free tuition classes*, *spoken English classes and then*, *in sports we cover indoor and outdoor*. *Outdoor is cricket*, *soccer*, *tennis*, *volley ball and things like that and indoor carom*, *chess*, *running and catching*. *It’s all simple*. *This is open for all*. *So*, *in indoor games*, *a lot of boys from youth club come and play with our own men [clients]*. *They play chess*, *they play carom and volley ball within our complex*. *They [local boys] also take our men to the beach and play soccer casually*. *They wouldn’t have a match but they would just play so that they get to interact also*. *[Through this approach] we are simultaneously [decreasing] stigma and we are also getting the men [clients] to engage with the community and also community developing in its own way*.

Staff Member

This inviting of communities in was another leveraged strategy. It was described as reducing stigma, educating the community about the work of the organization and opening doors in a way that helps staff better understand what the community needs, and raises awareness—and by proxy assists with fundraising and volunteerism. This is essential even at the most basic levels–communities came to understand that mental illness is something that the organization can help within a culturally and context relevant way. This was highlighted in the frame of prevention with some noting that too often "people will not come" into treatment or engage supports when prevention might still be possible.

*"Here we make four events—art expression*, *sports day*, *a conference and a round table seminar*…*we invite other societies to share in this*, *to exhibit art*, *to join us in our events*."

Direct Service Staff Member

Finally, there was a strong commitment to volunteers and volunteer development in these organizations. This was highlighted as essential in contexts where financial support was very limited. The purposeful blurring of boundaries between community and the organization was enhanced by volunteers—people who often had histories of helping communities and who by association enhanced the credibility of the organization.

*"If the government will not be able to provide these services in the future*, *we need to provide for our own community to help with problems at a community level*. *We were invested in improving our communities before this role [with current organization]*. *We always have been*."

Volunteer

Furthermore, volunteers were engaged in the organization culture—the culture of respect "equal say" and growth for everyone involved—again maximizing people's potential contributions.

*"I came here to volunteer actually*. *It wasn’t a decision to study and start work in a very structured way*. *So*, *I decided to work like as in art therapy before working with children which was logical as I studied art*, *so*, *I can teach them art with children with disability*. *That’s how it began but as I started work*, *I found out that I am kind of talented in this field*. *I know how to deal with them*. *I love them and all now*, *the supervisors told me that you can work in this field and you can start to study and just let it be your career*. *That’s how I started*. *So*, *I started to read and to have courses and of course to have training in the organization and now*, *I have just finished my Master’s in the field*. *That’s brief of how I started to work in this field*."

Direct Service Staff Member and Former Volunteer

### Partnership and Scaling

Two other subthemes, framed largely in interviews as being both a part of the process and as outcomes of leveraged ways of working were partnerships and scaling. Partnerships (developed after careful assessment to ensure the proper alignment of values and complementary activities) were emphasized as necessary to the provision of comprehensive services (e.g., plastic surgery for acid violence survivors), leveraging capacity (e.g., mindfulness meditation in Buddhist temples as an acceptable and accessible intervention), and in maintaining identity and focus (e.g., referring people with intellectual disabilities to a partner organization in that specific sector). Close partnership was essential to the scaling activities of all of these organizations—identifying collaborators who had the capacity to successfully implement and sustain the given intervention with fidelity.

More directly, the topic of scaling was very much on the minds of the leaders of these organizations though there was a considerable amount of variability as to the extent to which it had been undertaken.

*“Our strategy in scaling up is we set models*, *we study them*, *we fine tune them*, *we develop protocols*, *then we impact policy and through policy and advocacy*, *projects are scaled up*. *So*, *much of our models or aspects of our models have been incorporated into the mental health policy and urban health mission*. *That’s one part of scaling up*.”

Founder

In the context of scaling, the leveraged intervention was described as one that had been intensively examined and tested to identify the basic model—one that might be considered that of a set of ***"****minimum specifications****"*** or core approaches that are effective yet can be adapted to other settings.

*“Why do you think the model is so successful*? *It’s completely integrated*, *and flexible according to the local needs*. *There is no need for rigidity—it suits customs*, *culture*, *and universally applicable*. *It’s been proved in so many different countries*. *It aids with treatment*, *sustains livelihoods*, *and builds capacity*. *Sustainability is at the core of all its actions*.”

Senior Staff Person

Leverage and creating space or capacity were further evident in discussions about how scaling is best accomplished and, most critically, the infrastructure needed to effectively support it. As mentioned previously, some of this had to do with the selection of optimal partners and contexts for scaling. Other prominent topics here included a careful assessment of the resources needed to keep the expanded intervention connected and continuously assess fidelity and impact. As this work progressed the focus increasingly was upon "stabilizing" the resulting "alliance of organizations" through a network that supports continued growth, ensuring quality, and keeping people connected.

*“We think*, *the upcoming year we will also form almost 10–15 new groups for survivors*. *Within a year we will try to organize district level survivor conference where they show their problems*, *where they interact with government bodies and local authorities*. *We will give them a platform*. *That is a two day activity*. *That will create a district level network and we are trying to train them on basic leadership and fundamental leadership*. *When they get it*, *we will form formal network structure at district level and by 2 or 3 years*, *we have a plan to organize national level conference with all survivors groups*, *with district committees*, *with national committees*, *with the Public Prosecutor and Police*. *There*, *they will discuss their problems at national level and all that meetings will be facilitated by survivors*.”

Staff Member

### Points of Divergence and Limited Coverage

As noted previously, there was a great deal of similarity across the case study narratives with a focus upon non-specific aspects of developing and delivering interventions. The differences that did emerge across case studies were primarily ones of emphasis. For example, participants from some organizations spent more time discussing scaling strategies than others. There was some diversity in emphasis with respect to the role of government. While all discussed the importance of engaging government there were differences with respect to what was needed. This ranged from a need for a general approval of operations through to collaboration on policy changes that were key to the effectiveness of the intervention. Further, there were some differences in what was being leveraged from existing community resources. This again ranged from a reduction in barriers such as stigma to points of synergy in intervention (e.g., mindfulness meditation in monasteries). Finally, while inquired about, more detailed and nuanced information about the use of formal research and technology did not emerge beyond a general commentary about utility.

This multiple case study has focussed upon examining the non-specific aspects of internationally-recognized interventions addressing mental illness in MICs through a social entrepreneurship lens. In the past 30 years social entrepreneurship has become an important concept in understanding how to identify and support individuals and organizations that are effective in addressing social problems [9–11]. It grew to a large extent from the need to identify and cultivate sustainable, contextually established and relevant interventions/approaches that have large impacts [10]. While less commonly employed in the global mental health literature [7], it is relevant in that it is a framework that focusses on the consideration of *how* successful approaches to mental health-related problems are developed and applied (i.e., non-specific components).

Herein lies an opportunity to bring the focus to the science of delivery [5]—the structures (people, resources, and organizations) within which specific, evidence based interventions might best be applied. These structures are complex and are well-addressed through qualitative methods, particularly in this early stage of knowledge generation. Accordingly, this study employed one of the most rigorous research inquiries in this area to date [7] and focussed upon organizations and people recognized as socially entrepreneurial through Ashoka’s intensive selection and award process [10].

A coherent model emerged in this study that included interventions underway in multiple MICs. Cutting across a number of themes describing ways of working in mental health were the two themes of leverage and creating space in which to work. It is understandable that these two particular themes arose in this study as the most prominent. Mental illness in most MICs is characterized by severe resource and infrastructure limitations and intense stigma from individual to structural levels. In such settings, and considering the complex nature of mental illness, leverage is necessary to maximize the impact of limited resources—through both obtaining a maximal output of resources and applying them to points in the complex system of mental illness where the least pressure supplies the greatest change. A leveraged approach, however, must be matched by systems and settings that can accommodate it. Effectiveness cannot be achieved if the problem is not seen as important, the service offered is not seen as relevant, and stigmatizing beliefs and policies limit actions and opportunities.

Underlying these themes of leverage and creating space to work were several subthemes. These included leaders who have developed a very deep and nuanced understanding of the mental health problem that they are addressing and the systems within which it occurs. They also are extremely capable in engaging and aligning diverse stakeholders and assessing and maximizing the potential contributions of strategic collaborations, staff, volunteers, community, and service recipients. Key points of leverage in this context included attention to livelihoods through approaches such as social enterprise and the many benefits that attend the empowerment of recipients in a manner that reflects key aspects of recovery-oriented care [27]. Complementing leverage, creating space in which to work was an effort that involved establishing and marketing a “brand” that is their organization, values, and way of working. This aligned with the key aspects of successful brands highlighted in marketing literatures. Namely that successful brands are distinct (applied in a way and place where other options are not available), are relevant and important, are esteemed by customers and fulfills their promise, is understood and familiar, and is innovative and dynamic to suit changing needs and conditions [28]. Branding, in these case studies, was greatly enhanced through connections from local to global levels with other prominent organizations and being recognized for the provision of high quality education to a range of sectors (e.g., government, legal system). In synergy with brand development was an intensive effort to generate social capital through strategic partnerships, engagement of the media and influential public figures, engagement of government, and embeddedness in communities.

All of these activities created systems in which a common language for the problem was cultivated, its importance agreed upon, and their organization positioned as having the solution. This was closely attended by efforts to reduce stigma and create policy-enabling environments. This set of subthemes attended both local and scaled initiatives, though the latter involved greater attention to the careful assessment of sites that could successfully support scaling and the infrastructure necessary to develop and sustain scaled operations [1]. Contributing to the evidence behind social entrepreneurship as a coherent framework, these themes overlap with those of another multiple case study of seven leading organizations operating in impoverished and marginalized contexts, none of which had mental health as a focus [29]. The areas of overlap included local capacity building that mobilizes existing resources, with an emphasis upon their being “systemic learning” organizations in which continuous cycles of education and contribution by staff and clients is central, and extending impact through alliances.

While helpful in some respects, the lens of social entrepreneurship is problematic due to its breadth and lack of specificity [30]. Similar to the problems that attend defining “recovery-oriented care”, it encompasses many domains and activities and applies to both individual and group endeavours and characteristics. Perhaps a better way to capture the work of the social entrepreneurs studied here, and the non-specific aspects of their work that are crucial to effectiveness and scaling up, is through a systems dynamic framework. This is highly relevant to the wicked problem nature of mental illness in low resource settings, in which the problem is typically poorly identified, changing, has no definitive solution nor a clear set of possible solutions, is a cause of other problems and a symptom of other problems, and can be explained in numerous ways [31].

Systems dynamics theory is largely present other fields (e.g., economics) though is increasingly being applied in areas such as public health and sustainable development [32]. It is premised upon the need to consider wicked or otherwise complex and dynamic problems in holistic, non-linear ways [33]. It is highly relevant to GMH in that the debate about specific versus non-specific aspects of intervention [2] is at least to some extent a debate about perceptions of a linear “project” approach being applied rather than a sustained system behaviour framework. Similar to the findings of the present study, system dynamic approaches emphasize the importance of finding leverage points in complex systems where small shifts can produce large amounts of change [33–35]. The participants in this study, whether systematically and/or intuitively, had located important points of leverage and were pushing them in the right direction. Less well-recognized is the premise that leverage in complex systems must often be matched by an “essential complementary activity” just as subsidized low income housing needs to be matched with job creation to achieve the desired impacts [33, 36]. In the present study, this was the observation that leveraged approaches were matched by a range of complementary activities designed to create space to operate and build social capital that enables the intervention. This proposition supports the conclusion of Kirmayer and Pederson [2] that a balanced approach to inquiry and practice of this type will likely have the greatest overall impact in the lives of those suffering from mental illness in low and middle income countries. This work would involve an improved articulation and means of measuring non-specific aspects of intervention *within which* the more circumscribed and trialed interventions are optimized. Indeed, this is a line of inquiry already in development as models for effective community engagement and social inclusion are being examined as core non-specific aspects of scaled mental health initiatives [37,38]. The findings of the present study contribute to this emerging area of inquiry and implementation science that attends it, albeit with a somewhat broader frame (e.g., consideration of leadership characteristics, engagement with, and conceptualization of the problems faced; facets of social capital generation such as branding).

This study had a number of limitations. These included questions of representativeness beyond the organizations studied, a limited ability to comment upon how certain activities were directly causal of observed benefit, and a lack of an ability to compare these exceptionally successful interventions with those less successful. Considerations of component impact might be complex and require further study. For example, while some approaches to address livelihoods such as social enterprise have been associated with improve mental health and quality of life [39] and BasicNeeds and its livelihoods emphasis has demonstrated benefit [40], other models such as microfinance might negatively impact mental health [41]. Another limitation of the present study was that selection bias in participant recruitment might have been a factor as it is not possible to assess the degree to which past staff, key partner, and service recipient groups were fully representative. Future work, allowing for more fulsome comparison of more and less successful organizations aided through a random recruitment strategy would help to address these shortcomings, though the voluntary consent required by organizations to open themselves up to research scrutiny may always results in some volunteer bias. Also informative would be the examination of how these approaches might best be propagated through sharing information and mentorship—with propagation considering formal scaling, local growth, and sustainability through successorship as founders retire. Lastly, the scope of the interviews limited the ability to examine how specific interventions (e.g., supplying psychotropic medications) might interact with the non-specific elements highlighted in the study.

## Conclusions

In sum, this paper suggests the benefit of using social entrepreneurship as a framework for identifying successful efforts and the possible benefit of applying a systems dynamic lens to problems in GMH. It further suggests the potential benefit of matching the evidence base of circumscribed, specific interventions [3] with an equally rigorous inquiry into the non-specific factors without which those specific interventions cannot be successfully implemented nor sustained. Such work would assist in a stronger ability to identify and support promising organizations and leaders in the field and allow for the testing of methods for enhancing non-specific resources and strategies. This could lead to fewer failed endeavours and improved effectiveness and scaling of interventions. Indeed, as difficult to define as it is, social entrepreneurship in such a program of work provides a framework for considering how these very similar efforts at social capital generation might become better aligned to generate collective impact [42]. For additional quotes underlying the themes described in this paper please see S2 Appendix.

## Supporting Information

S1 Appendix Interview Protocols. (DOCX)

S2 Appendix Thematic Data from which Study Quotes were Selected. (PDF)

## References

[1] Eaton J, McCay L, Semrau M, Chatterjee S, Baingana F, Araya R, et al Scale up of services for mental health in low-income and middle-income countries. Lancet 2011;378:1592–1603. 10.1016/S0140-6736(11)60891-X 22008429

[2] Kirmayer LJ, Pedersen D. Toward a new architecture for global mental health. Transcult Psychiat 2014;51:759–776. 10.1177/1363461514557202 25358524

[3] World Health Organization. mhGAP intervention guide for mental, neurological and substance use disorders in non-specialized health settings.[Online].2010. Available: http://www.thehealthwell.info/node/69810. Accessed 27 August 2015. 23741783

[4] Bracken P, Thomas P, Timimi S, Asen E, Behr G, Beuster C, et al Psychiatry beyond the current paradigm. Brit J Psychiat 2012;201:430–434. 10.1192/bjp.bp.112.109447 23209088

[5] Kim JY, Farmer P, Porter ME. Redefining global health-care delivery. Lancet 2013; 382:1060–1069. 10.1016/S0140-6736(13)61047-8 23697823

[6] Wampold B. (2010). The research evidence for the common factors models: A historically situated perspective In Duncan B, Miller S. Wampold B, Hubble M,(eds) The heart and soul of change: Delivering what works in therapy (2nd ed.). Washington, DC: American Psychological Association.

[7] Kidd SA, Kerman N, Cole D, Madan A, Muskat E, Raja S, et al Social Entrepreneurship and Mental Health Intervention: A Literature Review and Scan of Expert Perspectives. Int J Ment Health Addiction 2015; advance access.

[8] Kidd SA, McKenzie KJ. Social Entrepreneurship and Services for Marginalized Groups. Ethnic Inequal Health Soc Care 2014;7:3–13.

[9] Drayton W, Brown C, Hillhouse K. Integrating social entrepreneurs into the “health for all” formula. B World Health Organ 2006;84: 591. 10.2471/blt.06.033928 PMC2627441 16917639

[10] Bornstein D. How to change the world: Social entrepreneurs and the power of new ideas. New York: Oxford University Press, 2007.

[11] Shaw E, Carter S. Social entrepreneurship: Theoretical antecedents and empirical analysis of entrepreneurial processes and outcomes. JSmall Business Enterprise Dev 2007;14:418–434.

[12] Available: www.ashoka.org.

[13] Available: www.skollfoundation.org.

[14] Cukier W, Trenholm S, Carl D, Gekas G. Social entrepreneurship: A content analysis. J Strategic Innov Sustain 2011;7:99–119.

[15] Stake R. The Art of Case Study Research. Thousand Oaks: Sage, 1995.

[16] Grisham T. (2009). The Delphi technique: a method for testing complex and multifaceted topics. Int J Managing Projects in Business 2009;2:112–130.

[17] Ritchie J, Lewis J, Elam G. Designing and selecting samples In: Ritchie J, Lewis J (eds.), Qualitative research practice. A guide for social science students and researchers. Thousand Oaks, CA: Sage, 2003.

[18] Pawson R. Evidence-Based Policy. Los Angeles, CA: Sage, 2006.

[19] Stake RE. Multiple case study analysis. New York: Guilford, 2013.

[20] Charmaz K. Constructing grounded theory. Thousand Oaks, CA: Sage, 2014.

[21] Available: www.basicneeds.org.

[22] Available:www.acidsurvivors.org.

[23] Available:www.thebanyan.org.

[24] Available:www.advance-society.org.

[25] Available: www.colectivoaquiyahora.org/.

[26] Deegan P. Recovery and empowerment for people with psychiatric disabilities. Soc Work Health Care, 1997;25:11–24. 9358596 10.1300/J010v25n03_02

[27] Slade M. 100 ways to support recovery: a guide for mental health professionals. London: Rethink, 2009.

[28] Mizik N, Jacobson R. The financial value impact of perceptual brand attributes. J Marketing Res 2008;45:15–32.

[29] Alvord S, Brown D, Letts C. Social entrepreneurship and societal transformation: An exploratory study. J ApplBehavSci 2004;3:260–282.

[30] Peredo A, McLean M. Social entrepreneurship: A critical review of the concept. J World Bus 2006;41:56–65.

[31] Head BW. Wicked problems in public policy. Pub Policy 2008;3:110–118.

[32] Paina L, Peters DH. Understanding pathways for scaling up health services through the lens of complex adaptive systems. Health Policy Plann 2012;27:365–373. 10.1093/heapol/czr054 21821667

[33] Hjorth P, Bagheri A. Navigating towards sustainable development: A system dynamics approach. Futures 2006;38:74–92.

[34] Nguyen NC, Bosch OJ. A systems thinking approach to identify leverage points for sustainability: a case study in the Cat Ba Biosphere Reserve, Vietnam. Syst Res Behav Sci 2013;30:104–115.

[35] Georgantzas N, Ritchie-Dunham J. 2003 Designing high-leverage strategies and tactics. Hum Syst Manag 2003;22: 217–227.

[36] Forrester J. Urban Dynamics. Portland, OR: Productivity Press, 1969.

[37] Asher L, Fekadu A, Hanlon C, Mideksa G, Eaton J, Patel V, et al Development of a Community-Based Rehabilitation Intervention for People with Schizophrenia in Ethiopia. PloS One 2015 11 30;10(11):e0143572 10.1371/journal.pone.0143572 26618915 PMC4664267

[38] Fekadu A, Hanlon C, Medhin G, Alem A, Selamu M, Giorgis TW, et al Development of a scalable mental healthcare plan for a rural district in Ethiopia. Brit J Psychiat 2016 1 1;208(s56):s4–12. 10.1192/bjp.bp.114.153676 PMC4698551 26447174

[39] Roy MJ, Donaldson C, Baker R, Kerr S. The potential of social enterprise to enhance health and well-being: A model and systematic review. Soc Sci Med 2014 31;123:182–93. 10.1016/j.socscimed.2014.07.031 25037852

[40] Lund C, Waruguru M, Kingori J, Kippen-Wood S, Breuer E, Mannarath S, et al Outcomes of the mental health and development model in rural Kenya: a 2-year prospective cohort intervention study. Int Health 2013;5:43–50. 10.1093/inthealth/ihs037 24029845

[41] Lund C, De Silva M, Plagerson S, Cooper S, Chisholm D, Das J, et al Poverty and mental disorders: breaking the cycle in low-income and middle-income countries. Lancet 2011 378:1502–14. 10.1016/S0140-6736(11)60754-X 22008425

[42] Hanley Brown F, Kania J, Kramer M. Channeling change: Making collective impact work. Stanford Soc Innov Rev 2012;20:1–8.

