# Supplementary material for: A Multiple Case Study of Mental Health Interventions in Middle Income Countries: Considering the Science of Delivery
Source: PLoS One. 2016 Mar 24;11(3):e0152083. doi: 10.1371/journal.pone.0152083 (PMC4807053; doi:10.1371/journal.pone.0152083)
Supplement: S1 Appendix — (DOCX) [file pone.0152083.s001.docx]

**S1 Appendix. Interview Protocols**

Interview Protocol for Ashoka Fellow, staff and volunteers*

1. Can you describe for me how your program/service came into being? (here probe challenges and facilitative elements)
2. For all services offered: What services and programming do you offer your clients? How do those services work? What makes them effective? Who is involved in providing those services? What skill sets are needed to do this kind of work?
3. What is the role of management in this program? How are decisions made and communicated?
4. Who are your clients? (here develop rich descriptions of targeted client groups)
5. What are the contexts, community and otherwise of your work?
6. What partnerships do you have with other organizations, agencies, and communities? (here probe in detail the nature of these partnerships – types of relationships, shared aspects of work, flow of clients to and from, facilitative elements and difficulties)
7. What kinds of benefits do you see for your clients in your program?
8. What kinds of benefits do you see resulting from your services for the larger community?
9. What types of problems do you run into in offering services? (here probe difficulties at client, staff, service, community, service system, social, and policy levels) How have you addressed those problems?
10. What types of changes would need to happen to improve your ability to do the work that you do?
11. How would you envision expanding your service? ( here probe local expansion and potential applicability of service in other jurisdictions)
12. If you could go back, is there anything you would have done differently in the development of the services you offer and the organization as a whole?
13. In what ways do you use information technology? (here probe past use, current use, and the potential for development in the future in this area).
14. Any other areas that would be important to discuss?

*Note: This interview framework formed a starting point for conversations with participants which flexibly explored additional topics as they arose.

Interview Protocol for Service Recipients

1. How did you find out about intervention(X)?
2. How did you come to get connected with X? Were there alternatives and, if so, why did you choose X?
3. What kinds of services/programs have you engaged in with X? What have they been like (here probe helpful and not helpful elements, what could be improved, how they have evolved over time).
4. In accessing X what have your relationships with staff/leadership/other recipients been like?
5. How has X benefited you?
6. Could X be improved and if so how?
7. What about X do you think has led to (points noted in #5) improving?
8. What benefits do you think X has for the broader community?
9. If you were in charge of X, what would it look like? Would it be the same or different, and if so how?
10. Any other areas that would be important to discuss?

Interview Protocol for Key Partners*

1. Can you describe for me how you can to know about and become connected with X?
2. What is the nature of your relationship with X? Has that changed over time? (further prompts tailored to type of relationship)
3. Can you speak about the impacts of X (probes from individual to community and country levels)? What makes X effective in these areas? What hampers effectiveness? Has this changed over time?
4. In what ways have you supported the efforts of X?
5. Have you run into challenges in your relationship with X? If yes, can you describe them and what happened?
6. What have been turning points for X? How did those turning points unfold?
7. Is there anything X could have done differently over time to have more of an impact? Might your involvement with X have been done differently to improve it? How so?
8. What do you see as next steps for X and for your relationship with X?
9. Along with your own, what other important partnerships does X have? Can you describe them and why you think they are important?
10. Any other areas that would be important to discuss?

*Note: This interview framework formed a starting point for conversations with participants which flexibly explored additional topics as they arose.
