# Supplementary material for: A Multiple Case Study of Mental Health Interventions in Middle Income Countries: Considering the Science of Delivery
Source: PLoS One. 2016 Mar 24;11(3):e0152083. doi: 10.1371/journal.pone.0152083 (PMC4807053; doi:10.1371/journal.pone.0152083)
Supplement: S2 Appendix — (PDF) [file pone.0152083.s002.pdf]

S2. Raw Data File (note: identifiers redacted)

Key characteristics of being a SE in mental health:

Understanding people, treating people equally, being non judgmental, being down to earth, open minded, balance between rational and emotional, strategic thinker, a good communicator, trustworthy, setting examples by doing things, team building , team spirit, mobilizing people and resources. I have a dream that one day a survivor will be the executive director of [redacted]

On finding leaders:

[redacted]

what qualities do you think make a good fit for the leaders on the ground where these models are being implemented?

[redacted] It'll be very similar. I think it'll be very similar. It won't be precisely and absolutely the same, but they're going to have to have good leadership skills, they're going to have to have a dogged determination to work in a field, quite frankly, most people don't want to work in. They're going to have to have an ability to get into the office of the senior people in their region, district, or country to be able to make resources available.

They're going to have to be prepared for the long march. This is a long march, because A) it's a chronic illness, B) it's been almost untouched for centuries, you could argue since the Bible was written. This is a long march. People who are looking for a quick buck or being part of something which is a little bit trendy or who have misunderstood what a long-term implication this is will be making the wrong decision, and hopefully we'll be able to wrinkle those out at the due diligence stage.

[redacted]

A champion can be… Ideally, for me, it's a person from the country. It's a person with a good knowledge of the country, and who has innate ability to command attention, respect, and sometimes leadership, even from their elders which is important in this conversation and who has a passion to be ambitious in the field that you are interested in.

As you know perfectly well, to show too much ambition in the countries that border you and even perhaps in Thailand itself, can be considered vulgar. But yet, unless you have that quiet drive and can be ambitious in your own way and a culturally appropriate way, then you're probably not going to achieve the mission that you outline.

The concept for the ultimate goal is one of putting oneself out of business “If you ask me, I would love to see a situation where [redacted] is no longer there in [redacted] so that we can send the story of Acid violence into museum. I would love to see that as an individual and as a chair person of [redacted], that is my vision. At least I tried to inculcate these values into our colleagues [redacted]

It is a social commitment and it is more like a mission. I should say that as a human being, it is my duty, my responsibility, my obligation to the society. If we take in that way, we will find a way. The inspiration that the survivors have given to us, the courage and strength that the survivors have given to us, that gave us the spirit. Then, it is more like, small success gives us the strength to move further. Believing in humanity, believing that there are good people and we need to mobilize them. They need a platform and we have given that platform. Recognizing people is very important.

[redacted] **550**

It is difficult to say in one word but I think the key element in this is not to give up, have the commitment and patience, have confidence that we can address this issue. We often face, I did face the situation personally. We have done so much but it is still a problem. So, by giving up or by accepting the reality, we will be actually strengthening the hands of perpetrators. We don’t want to do that. We cannot do that. So, we must have the courage and capacity to really stand up to the violence or human rights and at the same time, do it together. This togetherness is important. To develop the ownership of all the stakeholders involved. Also, third component is to do it in a comprehensive way. There is no one intervention point that is key. [redacted] has multiplicity of intervention points. All are needed.

[redacted]

Further in describing target, there was described the value of focus, of concentrating on one issue and sticking to it: In all aspects of services that are provided, survivors should be at the center and survivor’s participation is very important. Also, we should not undermine their abilities. We should provide them with an environment and guidance. Empathy, equality, respect, dignity, if we consider all these things, then, you will actually empower them. I think it is important also that where there are no services available, then service is needed. Otherwise it is very difficult to push the government of what is possible. In a society where there is no model response, then, we have to show a model. Even if it is in a small scale, they need to show what is possible. When we started we had criticism, why we are focusing on a small issue like acid violence. Why not burn as a whole? Another criticism was why we are not addressing gender based violence as a whole. Why acid violence? And we said that it is such an issue which manifests all the aspects in each issue. If we consider this, if we are successful in this issue, then we can show the wider aspects. In that way, we concentrated on one issue. This is unique. I think

it is also very important to identify the like minded people, the organizations, development partners, media, and celebrities. These are very crucial to support in the cause.

[redacted]**, 510**

[redacted]**, 710**

SEs in mental health have a number of key characteristics – with this information derived from interviews with fellows themselves, their staff and colleagues and key partners. Some part of this has to do with beign "charismatic", which was defined an ability to "motivate and inspire people" ([redacted] This characteristic, or versions of this, were present across all case studies. Characteristics went well beyond charisma, however. A very broad and complex skill set and some key values were also present. Frequently cited were "passion", "sincerity", kindness, and a relentless pursuit of a "vision" and cultivating "transparency" with respect to their motivations and actions. I don't give up. I am a fighter. I don't give up easily. I have a vision and I work towards my vision and my love for my son and for other people.[redacted] **410** Along the lines of vision, was this notion of trust – cultivating trust and having integrity:

In terms of the strategies that they employed, all cited the importance of generating ideas and being will to take risks and experiment "being keen to learn and experiment more[redacted]). This did not only involve themselves, but permeated the culture that was cultivated in their organizations – which were not hierarchical – "allowing other people to take over" ([redacted]) . "My risk taking ability is huge and also, I am a strategic person and whatever I do, I like having fun doing it." [redacted]**, 375**

I think two things. I developed a culture and [redacted] agreed with me. I developed a culture in [redacted] from the very beginning where everyone was equal. It did not matter if it was an executive director or a ward boy. We all were equal and all had a contribution to make. That culture was very different to [redacted] culture.

[redacted] **75**

The theme of positivity was also present – generating hope, sustaining vision, and being able to assess where opportunities lie. "She is very positive and she will make others also feel positive. You don't feel negative in her presence. … She also has X-ray eyes, how to diagnose your efficiency or hidden talent to bring it to the world so that one can contribute to the world. ….She makes life very vibrant and her surroundings also like that."[redacted] **140**

The culture of their organizations were ones in which everyone was involved and had an important role in a shared effort – as leaders this was cultivated – these were not described as organizations driven by or overshadowed by one large personality.

For everybody here [redacted] even if it is a cook's birthday, a driver's birthday, that happens and most importantly, she doesn't discriminate people. So, those children are also invited to her place to have lunch or dinner. Clients from here are also invited to her place for a meal. We also have a movie time at her place where all of us, it is mix of children, housekeeping, drivers, everybody, we have a movie time together which means that it is not about who you are but it is about what you are to me which matters to her a lot. And she didn't let go of people very easily also. It is like, if she has rehabilitated somebody 10 years ago, she wouldn't see that as a 160th women who has been reintegrated. It is not numbers for her but the meaningfulness that she derives from that reintegration. [redacted] **600** The leadership skills are very important. An organizational leadership you know. It is not just one person taking the leadership. We have to make sure that each staff member at their own level is taking the leadership and doing whatever they can contribute. Equality is practiced in the organization and there is no hierarchy. We praise a lot. It is very important, the recognition of people. Whatever they do, small thing, like [redacted] whatever small achievement, it inspired us the same way, it inspired everybody together and we celebrated that and that actually helped us to move forward. [redacted]**,** [redacted]

[redacted] to leaders: But, whatever it is, underneath she is very emotional and she gives so much of importance to the residents. All the residents love her. They don't need anyone to come [redacted] maam and them. If there are any complaints, she see's that they are sorted out. That is the main thing. You have institutions where the institution head doesn't care about what's happening inside or doesn't talk with the residents or whatever. But that doesn't happen in [redacted] When she comes in, they come and talk to her. She goes and talks to them. That is the strength in running the organization and a huge credit from her.

[redacted] **215**

People in these organizations are encouraged to grow, and learn and lead: She always provides the department with all the books or different information or things that could support to the department. She gives the staff the opportunity to improve their skills and improve their work. She doesn't interfere with other people's specialty. She always leaves people to look after themselves, it means, she respects their specialty.

[redacted]**, 90**

Madam [redacted] used to help us all really and she used to concentrate on helping us improve our knowledge and getting more information, updated information by getting people from outside, abroad to give us the updated information about the special needs and everything. She used to push us to have courses and things like that. I took courses in reading, writing, OT and things like that, and then, when it came to studying, she really is the one who helped me with this and she pushed me to have the Master's degree from [redacted]. Hamdullah! I got it.

Wow!

Ya, and that's very sweet of her. She is really helpful with this. She insists that we improve ourselves in this area. From that on, I have the certificate now which is qualification in the special needs.

[redacted] **40**

Apart from democracy which is run by the mental health professionals, there should be a sociocracy, you know, where lay workers, auxiliary workers, community workers, caregivers, they also get a lot of information from us, power like that and cohesiveness as a group, team. So, that would come in there.

[redacted]

Key here is a strong ability to communicate with and engage a wide variety of people, along lines of power and culture: "Her personal skills, she knows how to be lovely, very organized and another times, she knows how to be like strict or firm in some aspects to take decisions. She is decision maker I think, yaani, she is good at this. And she has the ability to make lot of contacts with people from different cultures, yaani, she has the ability to do contact with office boy or the minister of something, yaani, and get benefits from all and how to deal with them and how to administer all together." [redacted] **0112, 425.** And, critically, they are able to facilitate diverse sectors getting aligned around their vision.

On understanding the problem

There was an intensity around how the issues/problems came to be understood – growing mostly from direct experience, a lack of 'professional distance' and intense compassion. This was critical in informing the strategy to be taken – it grows out of this deep and intensive understanding of the problem and the needs of the people, families, and communities therein. This did not always come from lived experience but at times was sought out strategically:

[redacted] and her son). "So, I started to travel to get programs. I went to [redacted] in 1996. It was a center which was set up like a family home and I liked the atmosphere. I liked that it was friendly and my son didn't feel that he was walking into the clinic which was a situation he didn't like and actually started very bad behaviors when we did." Positioning self in the problem, through empathy or through lived experience, leads to a recognition that a holistic approach is needed: I asked myself, if I was attacked with acid, what would I want? Firstly, I would need medical help and secondly when the medical help is finished, I would need rehabilitation. I would need help to reestablish my life. Thirdly, I would want justice and cost to deal with the attacker. Those were the three things. I then realized that if we have to overcome this problem, we need to have a campaign. So, [redacted] developed four strategies. Medical aid which involved the area that you are interested in and that is psychological support, emotional support, secondly legal rehabilitation and then the legal and prevention campaign. Those were the four strands of ASF and we developed from there." The approach builds from need – as understood from the inside.

[redacted] Founder

We knew that these are the problems and survivors are suffering and we had to tackle the problem. We didn't know how to do it, because there was no organization which was addressing this problem from holistic perspective in the [redacted] acid survivor foundation was the first organization and this issue was so new to us. The medical component itself is so complex. If we consider, the legal component also is very very complex in its nature. The rehabilitation is also not just helping the victim. We have to work with the community as well. We have to work with the policy makers. It was not a one way solution and there was no model as such. I say that in my experience, violence against women was dealt from the legal perspective. If we are able to persecute the perpetrator, we can prevent the case, not just acid case but any form of Gender based violence and also, we thought that in this way we are giving legal justice to the people. But, we are not thinking about the other rights of the victim. We are giving only the legal rights to the victim but in acid survivor's foundation, in the beginning, acknowledging all the rights of the survivors and trying to do something to meet these needs. [redacted]

So, as parents we got together and we had parents support meeting and we decided that what our children need is beyond what a center can do, we need a total NGO, we need life span services, we need awareness, we need advocacy, we need a lot of other things that's not there. We also need to train personnel, because the personnel who existed knew may be about disability but were not specific in their knowledge about autism and sensory issues and so on.

[redacted] 20

So, from time to time, we get information about homeless women, men and elderly but we take only homeless women. After we get notified, we rescue them, we bring them here, we evaluate, stabilize them medically because they may have medical problems. Then, evaluate their

psychiatric status, initiate their intervention and the intervention is acute care when there is high dependency during that phase. Once symptoms recover, they go to a medium dependency place. Ok. Then, you reduce them to a low dependency. Then, simultaneously, we also do vocational training, socialization, rehabilitation, recreation, pet therapy, other kinds of psychosocial interventions and in the process reconstruct where they are from and then send them back to their own community.

[redacted] **20**

Needs always seen as comprehensive, including the need for livelihood:
For me, as an individual, I have seen this whole thing and if the government were running the centers everywhere and all that, proper community neighborhood centers, integrated centers both for physical, mental and social entitlements, if the basic living issues, if that was provided in your neighborhood within a 1 to 5 km thing and the community workers, they know you, your family, your circumstances and all that, I think 80% of life style issues, living conditions issues, environmental issues all that will be cleared.

Attending to family needs as well: So, for the patients who were reintegrated, we developed this program of disability allowance of 300 rupees, a very small nominal amount but we recognized that helped the families a lot, increased the service utilization. It kind of supported the families to support the cost of travel which is a very very important factor.

[redacted] **100**

[redacted]

[redacted]

> Treatment for many, many poor mentally ill people is secondary to survival. Although in the West we might think that the two are uniform or equally important, I think to many people in developing world, the most important is: livelihood, survival, making a pay, trying to turn a penny for your family, and it’s only then that you feel you can present yourself to the medical practitioner and see what’s wrong with you. Also because they’re frightened that they will be charged, even if they won’t be. They think they’ll be charged, and so they’re cautious about going into the medical practice of one kind or another until they know they can pay for it.

Lived experience – saw selves as a burden:

[redacted]

[redacted]

I was 22 years old and had mental illness and nobody understood what was wrong with me and it shamed my parents, I was unmarrieageable and a burden to their house.

[redacted]

We felt isolated and ashamed

[redacted] 365

We give them live stock in previous years. You know in rural [redacted] having a cow, that is actually a huge help or we even have set them up with small stores or we have given them sewing machine, so they can do some work on their own.

Coming up with pragmatic solutions that play to people's strengths: [redacted]

Later we started making candles during Diwali festival. It was a success and we got 1000 rupees or something as a profit. We didn't invest anything. Later, [redacted] founder said that we will buy moulds. That was the first time we started spending money for vocational training. This was around 1999 or 2000. Because of the medicines, they feel sleepy all the time or be idle. So, my work was to engage them during day time in an activity. So, I took some new papers and asked them to tear them apart. So, they kept doing that and that's how I kept them occupied. Later, I taught them handmade paper. So, we gradually made paper covers. They were sold out completely. We then received an order for 1000 invitation cards for wedding and was very particular that it should be [redacted] handmade paper. So, we did started printing unit. Still, lot of people were sitting idle. So, I thought why can't we do [redacted] print. I went to the founder and requested her to sponsor me for 15 days. I went to [redacted] to know how they use it. I took the training and printed with whatever blocks I had and called the person from whom I have learnt. He was so impressed that he called 2 to 3 people from [redacted] we gave the accommodation in [redacted] and they gave one month [redacted] and then we started work on that. One day while we were traveling, we saw rokali [redacted] – small grinding rocks) for sale. I bought 10 of them. These people don't know how to stop. They have repetitive behaviors. So, I brought some flowers [redacted] and asked them to grind. So, I converted their illness to an advantage. The grinded flower can be used as a base dye for the cloth.

[redacted] 55

A [redacted] community member: Re: Social change. I don't believe in magic. You can make all the great statements about advocacy change and policy change … I believe about CB services. Sustaining and teaching ppl that they can manage from the

resources they have available to them. [redacted] is like a fairy, doing something small and do-able, not too ambitious, than something big with no effect.

[redacted]

My favourite bits, as it were, the capacity building piece, because that's where we deploy all the group building skills, what we call animation skills, and so on, most vigorously. I like the livelihoods piece as well. As you know, I have worked in [redacted] for a number of years, and we have a lot of people in the field of [redacted] working in the livelihood sector of our program - [redacted]

[redacted]

[redacted]

Now I have been living with MI for 20 years and I no longer have the seizures, I make brooms. I sell enough for about 20,000 [redacted]

[redacted]

I wasn't afraid of learning [a new skill] [or learning broommaking]. But I was slow and really shy. I was patient; I came every day to learn. Even when the weather was hot (37C) in the afternoon.

Every day I went; even in advance, and earlier. I cycled over on my bike, and waited till someone opened the door. I'd ask someone to teach me … I asked the teacher to gather the materials and sew. Once finished, I was too shy to show it – hid it, thought it wasn't good enough – then showed the teacher, but was told that I was very good. I was very happy.

[redacted]

We saw her progress. And that she could do this [broommaking]. It was satisfying seeing her return to find a useful purpose.

[redacted]

It's good that I can make income, even though the income is very low ([redacted] [redacted] / broom). I can sell them to the ppl in the village. They feel sympathy and buy from me. I can make one broom every 2-3 days. So I'm no longer a person who just eats and does nothing. I am useful and can do something that has value.

[redacted]

So she is not forgotten in society. She makes brooms and with that, has money and earnings so that she can buy what she needs to eat. She can go out and participate in community, at pagoda. She is taking / participating in Meetings and with her brooms – this helps relieve stress and deal

with thoughts and be part of community life. The illness is managed/decreased and gradually her health is better.

My favourite bits, as it were, the capacity building piece, because that's where we deploy all the group building skills, what we call animation skills, and so on, most vigorously. I like the livelihoods piece as well. As you know, I have worked in agriculture for a number of years, and we have a lot of people in the field of horticulture and agriculture working in the livelihood sector of our program

On connecting livelihood with stigma reducing:

To reduce that blind prejudice, you have to be able to demonstrate, it seems to me, that the mentally ill person can come back into the fold, can be able to contribute back to society. Of course, in the end, make a living or part of a living. That seems to me to be a really strong feature of what we offer. Although livelihoods is of course about income and all the rest of it, naturally it is, it's as much as anything else about people recognizing the value of those individuals once again, maybe, or once and for all. I think that's really important. That's why I like that particular module anyway.

Later, we put a proposal to [redacted], we want to start a proper vocational training. How much can you support? Thus, we started proper vocational [redacted]. We have lot of volunteers and they themselves buy. Our gift wrappers got very popular in [redacted] along with the [redacted] prints also, 15 years back you can imagine. We had donor's core committee meeting. So, [redacted] to come and give orientation. So, they all came at 1pm. I brought all the products and sat on one side. They had meeting and discussed something. At 5 pm, my turn came and I gave presentation. They appreciated it. Next day, one of the [redacted] was impressed and gave two and half lakhs donation [redacted] I never expected that. I was so happy.

I brought blocks from [redacted] I also brought paper machinery. I organized everything and left the job because I was not a professional. I am more like a home world person. I am not even career minded also. I am an amateur but with a professional touch.

[redacted] **70**

Actually a lot. I will try to remember every step we went through. Regarding the kids, of course we developed a lot of programs like how to do [redacted] means individual educational program for each child and this is how we work here. We do an individual program for each child in [redacted] So, how we specify the strengths and needs for each child, we as senior staff have trained a lot. How to look at the child, to recognized their strengths and needs accurately, so you can put an [redacted] and work for it. We developed this a lot. We developed the curriculums and assessments we used. Actually, we did our own curriculums in some areas. Everything is related

to each other. By time, awareness in the society is better than before. So, we started to work in earlier age. Now, we have kids like 1 year and 6 months.

[redacted] 50

Part of this idea of comprehensive care is placing severe mental illness on a spectrum, and engaging communities

When you reintegrate them back to their family, then you will know what are the problems they face there. So, you have high stigma in the community, you have high stigma in that particular family itself. They don't know where they have to go, so access of treatment is another issue and even if they have access, their quality of care is a big issue. So, even if they have treatment, what happens to their functionality? Whether they have any employment opportunities? What happened to the family burden? Who is there to really support them? So, all these questions made us to think what our program should be. These are the learning's that we got from this group (homeless mentally ill women) we started exploring various aspects and now coming back and connecting these dots to really give them a proper intervention package so that we can prevent somebody from going into mental illness or prevent them from being homeless at least.

[redacted] 165

Along with comprehensiveness, is flexibility based upon client need (pure recovery oriented care): We can customize based on their need, based on their culture and based on their resources available. So, that is how we are working.

[redacted] 220

All the survivors don't have similar needs. Survivor who is victimized by her husband, a child who is victimized by her father, they have special needs and we have to consider them. They are most vulnerable class of our society.

[redacted], 85

The pharmacotherapy is very essential but apart from that, there are lot of psychosocial interventions which is being needed and that is not only for the clients and that we need to work with the caregivers and the family to make them also accommodate this person, to give them a

proper environment to really come out of this illness. It's not only related to their own body, not only related to their own mind but it is related to their environment and their people. So, we need to look into, ok, the client is better now and if he is functionally well, so, what is he going to do. Where is the employment opportunity for them? So, whose going to offer an employment? So, if they are not doing employment, again the family will say, you know, you are just being at home, you are useless and all those things and stuff. So, it's a chain.

[redacted] **390**

I think we need all our personnel's to be evangelical about mental health really really. I would not look at bland technical presentation but really engaging, to have those skills to reach the public. Beyond that, we should not only look at major psychosis that we have been helping because that is the one which leads to maximum disability but we also need to look at the whole wider spectrum of mental health and be aware because that's where the challenge lies as far as community health is concerned. When we do a community mental health program, we will not get that many psychotic or even psychotic person who has that much need that people in Banyan have. So, you will need to do a slightly different kind of work in a community setting. So, if we are looking into going more into the community, so this transit care is very focused in treating them and again getting them back to the community. So, you need to really work with the community, so that their awareness goes up, their participation in our program goes up and our skills there have to grow. How to handle the crowd, how to present this thing, understand the whole gamut of web of care, the care net that you could put around societies and how all of this have to work with each other. So, possible here, the inter professional rivalries are not much but it does have a certain space you know and even within professions, they may have inter this thing. Like I said, for me, it is not only Banyan but mental health and beyond that is comprehensive care and everything. So, if all of our personnel's also get that, you know, we can work together and learn from each other.

[redacted] **460**

But not always grounded in lived experience as [redacted] where lived experience was lacking they created it:

[redacted] first saw homeless woman on the street near their college. So, she was not wearing proper clothes. So, they borrowed clothes from a nearby house, gave it to her, they picked her up and left her in an organization who takes care of such people and told them, we will come back and see her in a week. When they went back a week later, she was not there. So, they felt that there is not proper place for mentally ill people like her. So, that's how they started. Initially, it was small and they were doing everything, even picking up from roads was done by them. Everything was done by them and slowly it developed.

[redacted] **20**

[redacted]

the defining moment was seeing a man in a cage being prodded by sticks, and that was the crystalizing moment where you felt mental illness was something that needed to be tackled.

[redacted] and we had decided to have a child, so we were going to have a urine test at a local hospital in [redacted] We came out of the laboratory with a [redacted], which is what we were looking for. There was a great big cage, and in the cage were these mentally ill people and they were being prodded. This was when I was in my very young 20's

[redacted]

So, [redacted] with them. So, it is not an organization run by them. They actually lived with them, they really understood what are the drawbacks that they had, what their needs are and it is all client oriented, I would say. If I want Dosa today, it will be like, I will get you that but what will you do to get that Dosa. So, if you technically see, this is a reinforcement mechanism but at that point of time, it was not being done scientifically. It is an immediate response for whatever we do. It is a more, I wouldn't say a charity based approach but it was more of a friendly based approach. But if you technically quote like, it is a strength based approach, client based approach, we can technically call all those things. So, that is how [redacted] originated.

[redacted] **60**

Initially, though they were the founders, they didn't mind doing anything. They have cleaned the bathrooms, they mopped the floors, and they used to cook in the kitchen, they used to clean and wash the clients, they themselves went for rescues, they themselves used to serve food. They have done the maximum from the beginning. So, it's not like founders. They have done so many things from the ground level and that made them to grow higher and earn so much of manpower through the services that they have rendered to the clients.

[redacted] **210**

This was a very personal investment – of own time and dollars -

On investment in the problem – sold their gold: We didn't have money at that time. [redacted] and [redacted] they sold their gold to pay the advance for providing food to the clients. After [redacted] and other organizations sponsored food, we could spend less money.

[redacted] **15**

So, while they have the ability or leeway to move around freely, it's also that especially with [redacted] who would get to them very personally in every way, really sit with them, sup with them, play with them, talk to them and everything.

[redacted] **95**

On looking after the caregivers – could link here with Ashoka'

In house capacity is one thing: So, they were providing some sort of emotional support. The problem was who will supervise them? Who will look after their emotions? They are also survivors. But by that time, the clinical psychology department established and students were coming out from there. Then, in [redacted] we hired a [redacted]. Before that we had a clinical psychologist who was supervising our peer supporter and then, we were sending our people who had difficulties, we were sending them to clinical psychologist for counseling. Then, we had our own department in [redacted] where we had one clinical psychologist, one supervisor from clinical psychology background and two peer supporters. So, by [redacted] we had an organization structure.

Personal resilience:

, it may not be the recovery that you or I conceptualize it but there is a state of personal recovery and I think many of them seem to be content with it. But, sometimes when it is treatment resistant schizophrenia for example, or the symptoms don't remit or the person doesn't get better or where there is co-morbidity or where there is intellectual disability with a high level of disability, then, it does anguish you, it does bother you, it does demoralize you because the person is not getting better, you develop your coping systems which is a part of the discipline that I come from, you are taught to build your resilience through reflective practice and through action learning and if you don't do that there is a tendency for, but that is also true for many entrepreneurs that if you don't build your resilience, you don't take risks and you are comfortable in an environment where there is lack of clarity of outcomes. But we are trained in our discipline, in our profession as entrepreneurs trained in the field somehow cope with it and build our resilience and try and celebrate those smaller joys and accept the lack of clarity. So, that's how we deal with that. So, that's at the client level.

[redacted]

[redacted] 280

Although people come from different background, all these people are working here because of their personal interest. They are like no matter what, I would do it and of course when some clients are aggressive at times, they might beat the staff members. So, now, we have support group for the staff. We teach the health workers how to handle such clients. Staff members also have debriefing sessions now and them. So, they can always come and tell their problems. We also provide separate counseling for staff for personal or professional problems.

[redacted] 405

On moving on: a few themes here, at [redacted] becoming more like "board members", Monira moving on to take the model into mental illness – but the organizations continue – this idea of sustainability

I feel that the [redacted] can operate without me and [redacted] That's our biggest strength today which means there is some level of sustainability because we have a very dynamic team that echoes our values and strategically also, live up to the expectations of both the founders and the board and the people that we serve. So, that is one organizationally.

[redacted] 390

[redacted]

Along with risk taking, there was a rigorous approach described with respect to expanding services:
Our strategy in scaling up is we set models, we study them, we fine tune them, we develop protocols, then we impact policy and through policy and advocacy, projects are scaled up. So, much of our models or aspects of our models have been incorporated into the mental health policy and urban health mission. That's one part of scaling up.

[redacted], 270

On scaling – needing a proven model of min specs that are translatable:
[redacted]

Since [redacted] let's say to December [redacted], we've definitely not only hammered out the model, but we also demonstrated its scalability and its functionality in different cultures and different regions of the world. That's a [req] for any franchise. You can't go out to the public, as it were,

to the marketplace and say: “Here’s a franchise,” unless you’ve hammered it down first as the thing that you wish to offer. [redacted]

Since [redacted] let’s say to December [redacted] we’ve definitely not only hammered out the model, but we also demonstrated its scalability and its functionality in different cultures and different regions of the world. That’s a [req] for any franchise. You can’t go out to the public, as it were, to the marketplace and say: “Here’s a franchise,” unless you’ve hammered it down first as the thing that you wish to offer.

We think, the upcoming year we will also form almost 10-15 new groups for survivors. In this group, they are raising their voice. Within a year we will try to organize district level survivor conference where they show their problems, where they interact with government bodies and local authorities. We will give them a platform. That is a two day activity. That will create a district level network and we are trying to train them on basic leadership and fundamental leadership. When they get it, we will form formal network structure at district level and by 2 or 3 years, we have a plan to organize national level conference with all survivors groups, with all district acid control committees, with national acid control committees, with [redacted] and Police. There, they will discuss their problems at national level and all that meetings will be facilitated by survivors. This work is running through survivor’s group formation and group meetings.

[redacted] 145

As well, and in line with this, is the notion of not settling, of continuously building and being responsive:

Otherwise personally, raising resources was a huge challenge in the beginning and because you are entrepreneurial, you want to reach out, you have a larger vision and you want to take those risks. So, obviously, you are never going to sit back on your laurels. We can easily say, we have done all of this, we will stop now and we will just because we have our credentials, we are a large organization, we have written the mental health policy along with nine others, we have brought in homelessness into urban health mission by being a member of urban health mission. So, we have done many things and we can sit back but we are not happy doing that because we still see distress and it is both the value that drives us to respond to that distress because we are a responsive and a learning organization, we are learning from the client and we respond to the client’s needs and that’s how our growth has been. It’s been the cycle’s that have been defined by the client’s basically. So, it has been user centric basically. That’s our core value to be user centric and to be responsive. So, while there are signs and everything else, this is what has driven every cycle and every growth. So, it has been organic in a sense.

[redacted] 225

Our biggest strength and the reason we exist is because we continue to be responsive and we continue to be driven to the needs of the people that we serve. So, I think that the passion

emerges from that. Because we want to solve problems, because we want to respond to the needs of the person, reach out and be responsive, we are able to develop services. We are able to look at the macro picture. We are also able to look at the micro realities and link them. We are able to innovate. All of that. I would think these two are most critical. Our people and our values.

[redacted] 395

On scaling – components rather that the whole piece which might be too context dependent

The other part of scaling up is to work through civil society players get them to replicate but we are not following the franchisee model. We believe that we can share and capacity build but not follow the franchisee model. Towards that, we worked with two NGO's and replicated parts of our work in the north east and in [redacted] Now, we have collaborated, we continue to collaborate with [redacted] but we have collaborated with [redacted] initiative to focus on the scaling up part of it where we will work in different parts of India through a consortion that we have formed and in fact expand a little more work in parts of south east asia and south asia through a network called the [redacted] that [redacted] and those spaces. So, we are looking at scaling up by sharing our resources and expertise both within the country, that's of course is our primary focus but of course in south asian and south east asian region with these partners and look at it as a mutual learning sort of exercise. So, [redacted] in touch with us on this.

[redacted]

Scaling, fidelity and control, role of IP:[redacted]

The ownership aspect is that we still retain ownership of the property, of the intellectual property, but we also have the satisfaction of seeing that ownership dispersed in a controlled way.

The control aspect is important, here. Not just the sharing aspect, but the control aspect means that you have some possibility of managing quality, because in Bangalore, India, we have what you could call, I call it the control unit. It's actually called our policy and practice directorate under Ashoka, but it's essentially a statistical department which collects statistics not only from our programs but also now from our franchisees. That's what gives us the indication in the detail of the statistics as to whether a program is willing to move ahead and make the effective impact it should do, or whether it needs some further controls. It will allow us to dip in and out of the social franchisees relatively fast. We won't lose all control. We might lose all ownership of the IP. We will have a reasonable jurisdiction in a world which is not completely perfect. We will have reasonable jurisdiction over the property that we first designed.

[redacted] Reasonable ownership over the property you've designed, but still with the room for the model to grow in the sites where it's implemented.

[redacted] Absolutely. I think you're right, because that's been… Flexibility and growth. Flexibility and interpretation of the model is very important.

[redacted]

Why do you think the model is so successful?
It's completely integrated, and flexible according to the local needs. There is no need for rigidity – it suits customs, culture, and universally applicable. It's been proved in so many diff countries. It aids with tx, sustains livelihoods, and builds capacity. Sustainability is at the core of all its actions.

And research and collaboration and evidence generation – this helps "prove" what we're doing. Most NGOs have diff ways of working and addressing these things – but [redacted] are catered to v systematically and comprehensively in the model.

Also the bottom-up approach, with full range – local ppl, they have their say; right up to gov't policy. From micro to macro.

On scaling and the structure to support it:

[redacted]

Scaling up: Seven essential features

Features:
1. Community-Focussed government policy
2. Dependable funding
3. Local mgt structure
4. Appropriately adequate HR
5. Active user & caregiver participation
6. A network of stakeholders
7. Local context adaptations

[redacted]

we have moved from being a single cell organisation, [redacted] with its international programs, and we now have become a multiple cell organisation called "[redacted] (not a formal name). We have trustee boards managing [redacted] That's half the program structure coming off the

books of [redacted] so the risk is reduced in terms of [redacted] and the organisation is as it were in a number of different countries.

[redacted]

The big vision, the big attraction, the next big importance for us is to recruit and stabilise into the alliance of organisations that we now create is to take in over the next period until December 2018, a five-year program, is for us to take in up to 40 social franchisees.

On scaling – building on local initiatives, setting standards and the dilemma about whether or not to build externally:

[redacted] 2002 when I took over the executive director position and [redacted] because his wife was posted in Uganda and he found the same problem there. He facilitated the professionals to form [redacted] was the second organization but by that [redacted] to mobilize people in [redacted] and then he organized [redacted] in [redacted] but when he found that it is not just a problem in [redacted] he came across some people working on this issue in [redacted] to make it more international and global. So, [redacted] is actually the initiator or contributor in forming all these organizations but all organizations are independent. People in these organizations came to [redacted] to learn how it works. He used the experiences in [redacted] in facilitating them in formation of [redacted] in respective countries. We had an international conference to develop standards for psychosocial support [redacted]

[redacted], 470

So, we said, what should be our strategy to move forward in the next 10 years? Should it be to set up more [redacted] like structures and continue with the number or should we be the one stop shot that defines what mental health care means for poor and homeless people and that by definition is 70% of India's population. Because if you look at 37% of our population, they fit into the category of below the poverty line today. It was lot more at that point. But, if you look at 70% of the population, they still earn only two dollars or lesser. So, really, you are saying that you want to reach out to the poor and homeless people, then, you want to cater to 70% of our population. Now, obviously, we are not the state. We are not the primary stakeholder. We are not the primary care givers. But, what can we do strategically? So, here we have applied our strategy. So, it was a combination of science which is what the problem is, assess it and what you need to do. And two, strategy. Let's then set strategy where we develop models, models that represent a comprehensive set of services for those group of people, showcase it to the

government and other civil society organizations, encourage to replicate and then use evidence that emerges from these models to impact policy. So, that's what we exactly did over this period of 10 years. We developed rural and urban mental health program across multiple clinics through multiple collaborations because we believe that there is no one way or one size fits all, so that's increasingly clear. The public health system, with the state government, with the rural welfare department through the [redacted] collaboration which is a world bank supported project, through corporation, through educational institution, multiple urban and rural programs and as we engaged in non-institutional care and we figured that in these clinics, the social care component had to be developed more strategically and greater science needs to be used in that because the interactions between the poverty and ill health or the environment and mental ill health is significant and now, you have theoretical constructs that also define mental health in those forms. So, those aspects we probed and thus began our journey of developing a social care program which focused on housing, familial care, poverty and children's education and multiple other aspects that you would cluster under the broad category of multi dimensional poverty. So, all these, lack of access, this is cycling which I keep explaining, where you were born, based on where you are born, your trajectory is pretty much determined. You have problems with nutrition, as a result, schooling, health access, marital status, your employment, all that is effecting, and thus the quality of life and thus the well being. So, that's the cycle of interaction between the environment, between your access to resources and mental health or between stress and mental ill health. So, we have decided to focus on that and we are currently in a stage where we are trying to define what exactly social care means when all these clinics were set up.

[redacted] 135

On the process of scaling – becomes less linear and more complex: [redacted]

> once you have quantum, once you have mass or the number of different organisations that all belong to essentially one kind of thing—let's call it the alliance—you then are able to generate what is essentially a second generation movement, which is essentially about conferences, workshops, the bringing together of all the [redacted] model for mental health organisations in India, blah, blah, blah.
>
> There's a kind of generational aspect which we need to add into the business plan which makes the business plan much more exciting and makes it much more less linear, and more and more chaotic. But I use the word "chaotic" in a very positive sense, just as one might use the word "destruction" in a very positive sense. Do you see what I mean?
>
> It's not linear and it enjoys very simple unconference aspects of complex theory. There is that complex theory that's attractive.

Turning points – resonate – getting government on board and becoming seen as an organization of survivors, and changing law: One is I would say, as it got ahead, [redacted] good support of both the media and the government in Bangladesh. The outside organizations are recognized as important powerful complementary force for the public sector initiative which is always there. But, on the other hand, it is perceived with a sense of skepticism as if it is like we vs them and that is prevalent for the whole civil society sector, NGO sector. But, in case of [redacted] it was very, relatively quickly possible to get the ownership of the government. It not that government actually support, we never actually got any substantial support but it was possible to create an opportunity to work with the government through different ministries like ministry of social affairs, ministry of home affairs who actually played a critical role in shaping the policies and there were two laws that were eventually formulated. This was one and it was complimented by the media. To be able to get the law, to be able to get the awareness in people and then actually move one. The second thing is that strategically as I said, it was very important that we looked at the organization as the organization of the survivors. So, the cause I would say was effectively taken to the people as demonstrated as an organization of the survivors. I think these are the two basic turning points to bring this organization to public notice.

[redacted] **65**

Less so a part of the story for [redacted] The major change is moving from a small place in [redacted] here to a bigger place in [redacted]. We wish the place here will become better and better. This will give us opportunity for better working with the student.

[redacted] **10**

On building support from key stakeholders:

On building something/compassion/hope in the people served – what was needed

So, [redacted] was the first survivor who spoke about her problem and she was not a direct target but she was trying to rescue her cousin who was the target. So, she was very badly burnt and she was also thinking that this is the end of her life but when she came to [redacted] and [redacted], I should say is very important here because she took not just an organizational effort but also a personal effort. She took this issue very seriously and provided a kind of guardianship to these survivors at the beginning who were completely hopeless, helpless and thought that they can never, they were not even thinking about surviving. They were thinking about committing suicide. So, this initiative, when the survivors found that someone is understanding them, treating them as real human being, respecting them, respecting their values and their thoughts and helping them to express themselves. That actually helped them more and more. So, [redacted] then started to talk with other survivors who were receiving treatment in [redacted] medical college hospital. In that way, drop by drop they were also growing.

[redacted] **45**

How organizations are structured: How people contribute

Being trust worthy from our own work. What we say, we do that. That is very important. Transparency and accountability. Honesty, integrity and commitment. Being wise, decision making ability, making connections, again the leadership. Motivational leadership I should say. It is not a one man show and we have done that. We have been able to involve staff members the volunteers, the trustees, the development partners, all the stakeholders. They found a platform and that was very important. When people find this equal share is there, they feel that they are contributing. We have challenges. There are some stages when we have anger and frustration and difficulties in many ways. But, we shouldn't give up.

The leadership skills are very important. An organizational leadership you know. It is not just one person taking the leadership. We have to make sure that each staff member at their own level is taking the leadership and doing whatever they can contribute. Equality is practiced in the organization and there is no hierarchy. We praise a lot. It is very important, the recognition of people. Whatever they do, small thing, like [redacted] whatever small achievement, it inspired us the same way, it inspired everybody together and we celebrated that and that actually helped us to move forward.

[redacted] 565

For everybody here [redacted] even if it is a cook's birthday, a driver's birthday, that happens and most importantly, she doesn't discriminate people like there are children clients who are here. So, those children are also invited to her place to have lunch or dinner. Clients from here are also invited to her place for a meal. We also have a movie time at her place where all of us, it is mix of children, housekeeping, drivers, everybody, we have a movie time together which means that it is not about who you are but it is about what you are to me which matters to her a lot. And she didn't let go of people very easily also. It is like, if she has rehabilitated somebody 10 years ago, she wouldn't see that as a 160th women who has been reintegrated. It is not numbers for her but the meaningfulness that she derives from that reintegration. She will still ask, how is this person who is reintegrated there? Out of the blue she will ask whereas we will take some time to understand, who this person is, where this person is. So, that amount of memory she has. And she will also talk about the person briefly if not completely, she will say, she was living in this place. I think she has these issues. How are those issues now? That will give work for another week to find out all of these things. So, that loop also, she completes. It is not like, though she hasn't come here often, she knows most of them here let it be their stories, let it be the kind of trauma they went through. Even case managers, once they say that they have got a better job and they left, when they come back, she doesn't say no to them. She is not so vindictive.

[redacted] 600

She always provides the department with all the books or different information or things that could support to the department. She gives the staff the opportunity to improve their skills and improve their work. She doesn't interfere with other people's specialty. She always leaves people to look after themselves, it means, she respects their specialty.

[redacted] **90**

[redacted] used to help us all really and she used to concentrate on helping us improve our knowledge and getting more information, updated information by getting people from outside, abroad to give us the updated information about the special needs and everything. She used to push us to have courses and things like that. I took courses in reading, writing, [redacted] and things like that, and then, when it came to studying, she really is the one who helped me with this and she pushed me to have the Master's degree [redacted] and [redacted] Hamdullah! I got it.

Wow!

Ya, and that's very sweet of her. She is really helpful with this. She insists that we improve ourselves in this area. From that on, I have the certificate now which is qualification in the [redacted]

[redacted] **40**

and I try to push the staff to continue their education to go for a masters degree, to go for a PhD not only because of the degree but because of widening of their thinking, just the process of doing the research of studying, and when I travel, when we have money, I try to take staff with me, so they will know. They also get the exposure, and they understand more, how things are happening, specially regionally, what's happening here, what's happening there, so they can compare and find out how can we do better or how can we change things.

[redacted] **255**

On being rights based as a fundamental frame – which helps to clarify who should be involved:

Little bit. We are more focused on right based approach basically, identify the survivors who can maintain themselves, they can reintegrate themselves into their families and who can be a leader for others. It is not possible to provide lifelong services however your funding is. This is basically government's responsibility, social responsibility. This is not the only responsibility of [redacted] Rights based approach helps in making them independent. We have to stimulate all the government institutions and people in the society so that it becomes easy for the survivors to get their legal rights. They should have easy access to legal services.

[redacted] 65

The issue of violence against women, nothing will change. Men have got to be involved. It was a privilege and an honor for me to be able to allow to work in [redacted]

[redacted] 155

The reason I am telling all this is because when they come under illness or disability or whatever, not only medicines, not only other care, they need to know about their rights. Only if they know their rights, they will be able to ask. See, today [redacted] is there. Tomorrow it might not be there. They should know the law, they should know the right. Only then, they can fight in a government set up.

[redacted], 95

Part of the strategy, beyond getting recognition for the issue, is developing a brand or sense that they are the ones who can solve it – it is marketing:

We devised the plan and the strategy by way of which we have understood the problem and realized the magnitude along with few other collaborators and then, we developed responses which were much more strategic and less immersed in social sciences. So, it was a more branding strategy, more management strategy. How do I get my team in place, how do I position myself, how do I market the issue, not so much of the [redacted] but the issue.

[redacted] 90

[redacted]

A. Ppl also see us as a competent, collaborative presence who is considered an attractive partner to collaborate with.

[redacted]

There's no doubt about it that funders respond to volume, and so the fact that we have been able to raise volume in our current program in our programming has certainly evidenced it of course. Lots of NGOs say things like: "We work with millions of people in the third-world, and nobody, it seems, ever says, 'Prove it,' at least when they say it like that." Actually, if they had to prove it, they couldn't prove it because they don't have the system to prove it and it's probably not true.

In many cases, so in our case when we say we have transformed the lives of [redacted] people in [redacted] – we mean it. We are quite prepared to accept that as statistical variation of so many percent are up and down. Funders really appreciate that type of thing, and that seems to be in terms for them and it's important to me. In fact, it's just as much a part of the vision that we have a model of operation that works at volume as any other part of the vision, really.

[redacted]

A. We're a v prominent part of the global mental health community. And we have a place there. It's not a cakewalk – sometimes I get ignored around the table but we are starting to turn that around. Ppl also see us as a competent, collaborative presence who is considered an attractive partner to collaborate with. I hope we don't come across as arrogant … we are collaborators – you came to us -- therefore we can also teach you. - [redacted]

Part of this is quality "quality services" [redacted], and in [redacted] where quality is high and backed by evidence – something that people respect and can identify. Comprehensive and excellent quality – for the poor or not.

This includes capacity building – the idea that with a quality service, you can generate exposure and revenue by doing trainings: For capacity building, we have partnered with [redacted] society of burn injury and [redacted]. They are biggest services in the world and these join together and working with the government to improve the Burn care services. We have a training program which is supported by Inter burns, [redacted] charity. We do training based on their curriculum. They train our staff and then, we train [redacted] service care providers. Until few days back, the services for burn treatment were predominantly in [redacted] but now medical college hospitals of [redacted] we are training them [redacted] to maintain standards in treatment of burns.

[redacted] **45**

[redacted] has expertise to use experience for other gender based violence. [redacted] has to be strong evidence based organization.

[redacted] **225**

Now, we are working with the government project called [redacted]. So, it is also sharing of our own knowledge you know. We are working in [redacted], rural mental health program. It is a similar project that the government is trying to explore. So, our own expertise is the resource that they have got. We work as a regional resource agency for them to set up a community mental health project in a particular block.

[redacted] **225**

So, what we do is we also build the capacity of other organizations either can be governmental or nongovernmental organizations. So, we have done it with different NGO's across India. So, the [redacted] itself doesn't want to necessarily expand. We don't say that we will start [redacted] in [redacted] we will start [redacted], not like that we like to work with local organizations who have expressed interest working in same kind of area. So, then, we have a engagement with them usually for a longer period of time usually 1 to 1 ½ years longer where we visit them, they visit us, we understand the situation, they visit us to learn from our models. Then, there is lot of hand holding happening after that and they take it forward themselves. So, it is more capacity building where we ourselves are scaling up. So, that's a plan which we also would like to expand in future.

There is no Arabic curriculums for academic skills or speech and language or psychomotor or art in the Arab region and when we started working on it, we shared our experiences with some countries Saudi Arabia, Lebanon, Kuwait, United Emirates. The one who starts after that in [redacted] founding the [redacted]

[redacted] **175**

Mainly a fresh graduate one. He took an intensive training course here in [redacted] theoretical and practical under supervision of Mr. [redacted]. Then, the teacher starts his work with the child from his home to the school and from his school to home. Ok. Not related to the society, just in supervision under [redacted] with the teacher and the child, if they need any assessments, programs, any facilities, they need teaching methods, refreshing training courses, refreshing seminars, workshops. That's their role.

Ok. Basically, they train the teacher and they go into the mainstream and try to help the teachers. So, his role is mainly training.

Training and supervision of the inclusion process.

[redacted] **25**

[redacted] **80**

Part goes beyond training, to coordinating: [redacted] because it is the start of the outreach programs as an NGO. We started with [redacted] as the representatives of special education in the [redacted] module, Education for All. From that date, the [redacted] opens services to all over the [redacted] From that date, we start to build up the partnerships, from 2004. You can imagine that in those first ten years, a lot of networking, partnerships in works inside [redacted] and the region and internationally, starting from the internal networks from the NGO's in [redacted] to the regional networks to the international one's. That starts from 2004 with [redacted] and the governmental way and the other one, in several NGO's we are working on, we are not working on intellectual disability only. We are working on intellectual disability, developmental disorders and also learning disabilities and networking in those three specialties. Another way of inclusion, it opens the umbrella to all over the disabilities to network and cooperate with other NGO's which is their specialty in visual impaired or disabled or hearing impaired to work under the umbrella of inclusion together. All of these networking and all of these partnerships have been done in the first 10 years from [redacted]. And at that time, I started to work in outreach program with [redacted] to so and so and so on. That's it.

[redacted] **65**

Part of the brand is values as well: Strength number one is [redacted] values. Ok. [redacted] is characterized by its very very strong values. [redacted] ability to sustain its work despite difficulties, [redacted] ability to bring in convergence, [redacted] to focus on vulnerability and focus on needs of people who are marginalized. So, bringing in vulnerability, poverty, marginalization to the platform by providing care and [redacted] ability to innovate with respect to human resources and services.

[redacted] **280**

But, starting was not necessarily strategic at first: The idea wasn't to grow big. It was not a strategic effort but a passionate outburst. So, the plan, we didn't have a blue print. The plan was to work in a very low profile way and reach out to small group of people, make a difference and then exit, and we thought the number was going to be very small.

[redacted] 60

How clients are viewed and what is cultivated

A key aspect highlighted by [redacted] particular has to do with how the clients are regarded – very much from a strengths based perspective – which translates into the organization being a facilitator of resilience and empowerment:

One thing I think struck me very significantly and that is something that I always say inner strength of the victims provided to survivors by the staff members. [redacted]

First is empowering the survivors. Without empowerment, because of the nature of the issue, it is not possible for survivor himself or herself to continue. Empowerment could be anything right. It could be education, it could be providing survivor life skills or social skills or even just training on a basic employment. They should be given medical treatment, then providing them with counseling, then giving social and economic rehabilitation, giving them development support similar to what [redacted].

[redacted], 120

Discussed by the [redacted] was the idea that they are not patients but people "like any other human being"

Again this notion of reducing stigma through humanizing the problem: I don't think we can call them special needs. We all have special needs. I have special needs in something. You have special needs in something. So, we shouldn't label them as special needs. Everyone can do what they can do. We have right and left hand. Right hand can cut in good way than left hand. Right hand gives good support to left hand. In our world, the man who has many skills can give support to the man who doesn't have. If people can understand that we all have special needs, we don't have to advocate for these kids for inclusion."

[redacted] 140

There is clearly an emphasis upon client empowerment and involvement – one that goes beyond just cultivating individual resilience. People are empowered, become staff, and become advocates:

Even with resource constrains, [redacted] provides heartiest help to all the survivors. I think this is the best strength. We empathetically provide these services. Another strength I would say is [redacted] provides quality services so that survivors can not only get instant support but can rehabilitate to the society, so that they can survive, they can be a leader. Before attack, maybe she was a normal person but after attack she became a leader to the community. So, I think this is the strength of [redacted]

[redacted] **40**

[redacted]

- The [redacted] model's basic ethos = participation of ppl w MI. The way it works is v esoteric, conceptual, -- we wanted to make research participatory and tangible to ppl. Particularly bc we are working w a clientele w MH problems, but who are also disempowered bc of poverty, low literacy etc … Getting them involved was a challenge. They are not used to being asked to join in on things. But not really when you think about it when you think of animation and concsientisation technique[redacted]

B. **Seeing people with lived experienced engaged inspires:** Nothing is as inspiring as seeing the change in lives of ppl who have lived with MI. And seeing this person participating in groups, and working—that's indescribable. That's the best. That's the heart of the work.

This feeds into funding activities with stakeholders hearing directly from those affect and they can inform the design and refining of services:

One thing that really matters is the person standing up for themselves. All the survivors standing up for themselves are a huge factor in funding these changes because they are fighting for their rights. This makes people do want to do something for them.

[redacted] **130**

Our survivors. Day by day, they are growing themselves in different organizations. They are in media sectors, they are in corporate sectors, they are in government sectors. Our survivors are growing. Our peer survivors, they are inviting other survivors. They are becoming role model. The main staff of [redacted] are our survivors. I have seen that our peer counselors, how they grow up

themselves. At the same time, they are becoming activists, they are becoming policy makers, they are becoming role models and they are working in different NGO's. So, they proved themselves. Sometimes, our perception is they are helpless. I have seen so many survivors from whom I get positive energy. Ok. If she can do, then I can do far far better. So, I get inspiration from them and all of them are fighters. We need to increase that attitude or that spirit. Some of them are studying abroad. It is amazing to see how could they accept the reality, how could they expose themselves in front of everyone without covering themselves, how could they motivate others. So, it is a huge energy they have. They have lot of confidence which normal people don't have. It is place of development for me.

On peers and capacity building, [redacted]

[redacted]

Examples of successes:

[redacted]

- …"It became apparent that follow-up visits were not taking place as the CBWs were not provided incentive … The responsibility was subsequently taken on by members of the user self-help group. … '**We feel this work is better done by us because it is we who are sick and should therefore support each othe**r' [[redacted] Chairperson of community-organised self-help group] **… Village mobilisers are more familiar with the participants I their community, have a vested interest in the [redacted] project, have experience of treatment and are aware of the benefits of the project to participants' lives."**

[redacted] **130**

On who is the most important – all rotates around the survivors: The recognition that we were a family and everyone in the family worked together, and that included the survivors. We pulled the survivors first. They were the most important people. It was not me, it was not [redacted] it was not the Surgeons coming from the [redacted] It was the survivors who were the most important people in the organization. So, that culture developed and I think that contributed to the success of the organization.

[redacted] **80**

This is relevant to growth as well – the human element must be maintained:

I think with disability, it is very important not to grow too much. You need to keep the human element. I need to go into the school and know every kid in there. I can't walk in and they just be faces. It is important to keep this human element. And this why when I set it out, I said we can never have more than 200 to serve in the building. But then, it can be many other buildings which are, so this is like our cocoon where we are creating, and then it can be disseminated and

[redacted], 390

It is important. Peers always understand their feelings. So, when our peer counselors share with them individually or in a group, they feel free to share anything, their own experience. So, it is helpful for them.

[redacted] 55

Other than medical support, she received counseling training. The right based approach like fighting for rights in terms of her own case and others cases and [redacted], training on becoming a trainer. She had two operations mostly skin grafts and she received educational support as well.

[redacted] 50
